# Variations in morphology, physiology, and multiple bioactive constituents of Lonicerae Japonicae Flos under salt stress

**DOI:** 10.1038/s41598-021-83566-6

**Published:** 2021-02-16

**Authors:** Zhichen Cai, Xunhong Liu, Huan Chen, Rong Yang, Jiajia Chen, Lisi Zou, Chengcheng Wang, Jiali Chen, Mengxia Tan, Yuqi Mei, Lifang Wei

**Affiliations:** 1 College of Pharmacy, Nanjing University of Chinese Medicine, Nanjing, 210023 China; 2 Collaborative Innovation Center of Chinese Medicinal Resources Industrialization, Nanjing, 210023 China; 3 National and Local Collaborative Engineering Center of Chinese Medicinal Resources Industrialization and Formulae Innovative Medicine, Nanjing, 210023 China

**Keywords:** Plant stress responses, Biochemistry, Plant sciences

## Abstract

Lonicerae Japonicae Flos (LJF) is an important traditional Chinese medicine for the treatment of various ailments and plays a vital role in improving global human health. However, as unable to escape from adversity, the quality of sessile organisms is dramatically affected by salt stress. To systematically explore the quality formation of LJF in morphology, physiology, and bioactive constituents' response to multiple levels of salt stress, UFLC-QTRAP-MS/MS and multivariate statistical analysis were performed. *Lonicera japonica* Thunb. was planted in pots and placed in the field, then harvested after 35 days under salt stress. Indexes of growth, photosynthetic pigments, osmolytes, lipid peroxidation, and antioxidant enzymes were identified to evaluate the salt tolerance in LJF under different salt stresses (0, 100, 200, and 300 mM NaCl). Then, the total accumulation and dynamic variation of 47 bioactive constituents were quantitated. Finally, Partial least squares discrimination analysis and gray relational analysis were performed to systematically cluster, distinguish, and evaluate the samples, respectively. The results showed that 100 mM NaCl induced growth, photosynthetic, antioxidant activities, osmolytes, lipid peroxidation, and multiple bioactive constituents in LJF, which possessed the best quality. Additionally, a positive correlation was found between the accumulation of phenolic acids with antioxidant enzyme activity under salt stress, further confirming that phenolic acids could reduce oxidative damage. This study provides insight into the quality formation and valuable information to improve the LJF medicinal value under salt stress.

## Introduction

Agricultural production is seriously threatened by various environmental stresses, such as excessive salt^1^, pathogen infection^2^, drought^3^, and nutrient^4^, etc. It is predicted that crop productivity will decrease by 50% in many parts of the world to 2050^5^. Among them, excessive salt is becoming one of the most detrimental and diffuse environmental stress and retards seed germination, plant growth, and productivity^6^. Medicinal plants, which are important crops to resist and cure diseases, are also under the threat. Hence, the quality evaluation and quality formation mechanism of medicinal materials under salt stress should be paid attention.

*Lonicera japonica* Thunb., which is native to the countries of eastern Asia and widely distributed in most parts of China, is one of the most important vine species of the Caprifoliaceae family. Therein, the flower bud or initial flower is documented as traditional Chinese medicine (TCM) in the Chinese Pharmacopoeia (2015 edition), named Lonicerae Japonicae Flos (LJF), and it is known as ‘jin-yin-hua’ in Chinese. Recently, LJF has also been applied to suppress SARS coronavirus^7,8^ and influenza A viruses (H1N1, H5N1, and H7N9)^9^, which pose a substantial threat to public health worldwide. Besides, its traditional Chinese prescriptions, Lianhua qingwen capsule have been approved for the treatment of COVID-19. Meanwhile, it is also widely used as healthy food, cosmetics, and so on^7^. And the market demand is increasing day by day. Modern phytochemical and pharmacological studies show that more than 200 compounds including organic acids, flavonoids, iridoids, volatile oils have been identified in LJF^7,10^, and these compounds possess multiple biological activities. For example, phenolic compounds have been frequently reported to display a vital role in anti-aging, anti-inflammatory, anti-oxidation, anti-proliferative, etc.^11^. Products rich in flavonoids are also recommended to reduce the risk of chronic diseases, such as coronary heart disease, heart disease, cancer, and diabetes^11^. Moreover, iridoid glycosides are responsible for antibacterial^12^, anti-allergic^13^, hepatoprotective^14^, and other pharmacological properties. Iridoid possess neuroprotective^15^, anti-thrombotic, and anti-inflammatory effects^16^. The accumulation and synthesis of bioactive constituents mainly depend on satisfactory growing conditions. And salt stress can stimulate the synthesis of chlorogenic acid and phenolic constituents^17,18^, which are the main active compounds in LJF. Hence, it is supposed that moderate salinity might be able to improve the quality formation of LJF.

Although the quality evaluation of LJF has been reported previously^19,20^, studies on the quality formation of LJF under salt stress are still scanty. Multiple physiological and biochemical responses under salt stress have been monitored in a variety of crops and medicinal plants^21,22^; however, there is still limited information on the association of morphology, physiology, and bioactive constituents with salinity in LJF. To the best of our knowledge, the experiments of LJF under the salt stress are mostly executed with hydroponics or potted plants, focusing on the alterations of a single or one class of component concentration^17,23^, which lack ecological proof and practical value. Because the quality formation of TCM is affected by various factors in nature, while single or one class of constituents cannot completely represent the quality of complicated TCM. For these reasons, we aim to investigate the variations of morphology, physiology, and multiple bioactive constituents in LJF under different salt stresses; developing a simulation approach to predict the quality of LJF associated with salinity through field trials; and analyze the correlation between antioxidant enzyme activity and total phenolic content in anti-oxidative stress.

Up to now, there is no public report on the changes in morphology, physiology, and multiple bioactive constituents in LJF under different salt stresses (0, 100, 200, 300 mM NaCl, respectively). Here, the morphological changes of LJF during the growth process under different salt stresses were recorded; Physiological indexes including photosynthetic pigments, osmolytes, lipid peroxidation, and antioxidant enzymes activity were evaluated. Next, the total accumulation and dynamic variation of 47 bioactive constituents including 13 amino acids, 4 nucleosides, 12 organic acids, 12 flavonoids, and 6 iridoids were quantitated by ultra-fast liquid chromatography coupled with triple quadrupole/linear ion trap tandem mass spectrometry (UFLC-QTRAP-MS/MS). Moreover, multivariate statistical analysis was used to evaluate the quality of LJF. Partial least squares discrimination analysis (PLS-DA) and gray relational analysis (GRA) was employed to systematically cluster, distinguish, and evaluate the samples, respectively. Simultaneously, the correlation between antioxidant enzyme activity and total phenolic content in anti-oxidative stress was also analyzed and discussed. This study can provide insights into the quality formation of LJF under salt stress and novel clues for improving the quality of LJF.

## Materials and methods

### Plant materials and salt treatments

The experimental site was located in Medicinal Botanical Garden of Nanjing University of Chinese Medicine (north latitude 118° 57′ 1′′, east longitude 32° 6′ 5′′), Nanjing, China. The two-year-old major roots of *L*. *japonica* were excavated from Henan province in December 2018. The botanical origins were identified by Professor Xunhong Liu (Department for Authentication of Chinese Medicines, Nanjing University of Chinese Medicine); these plants were planted in plastic plots (50 cm height, 34 cm of top diameter, and 26 cm of bottom diameter) with approximately 25 kg soil (texture: loam; organic carbon: 36.6 g/kg; cation exchange capacity: 17.0 cmol/kg; pH: 5.0) and placed in the open air before salt stress treatment. A shed blocking off rainwater was installed when the salt treatment began, other conditions were almost the same as the opened-air environment.

*L*. *japonica* was allowed to grow naturally, until these plants were alive and germinated. Then four concentrations of saline solution 0 mM (control, 0 g/L NaCl), 100 mM (low salt stress, 5.85 g/L NaCl), 200 mM (medium salt stress, 11.7 g/L NaCl), and 300 mM (high salt stress, 17.55 g/L NaCl) were conducted from 24 April 2019 to 29 May 2019. The treatments were designed with 5 replicates at each concentration. To avoid the osmotic shock, the concentration was increased gradually until the set salt concentration was reached. The stress treatment was executed at a rate of 2 L per pot every three days. The flower buds and leaves were collected from 5 randomly selected plants in May 2019. And then cleaned with phosphate buffer saline (PBS), immediately frozen at − 80 °C for the subsequent experiments. Some for physiological experiments, some for quantitative analysis, and the rest were used as voucher specimens.

### Growth parameters

The growth process of *L*. *japonica* before salt stress was showed in Fig. S1. Leaf diameter and flower bud length were determined during the blooming stage. Plant height and fresh weight of shoots per plant were measured on the last day of harvest. The salt stress injury can be divided into five grades, as follows:

- Grade 0: no symptoms of salt stress;
- Grade 1: a few leaf tips, leaf margins, and veins turn yellow;
- Grade 2: about half of the leaf tip, wizen leaf edge;
- Grade 3: most of the leaves become yellow and fall;
- Grade 4: the branches dry up and the leaves fall.

### Extraction and assay of pigment

The contents of chlorophylls and carotenoids were evaluated according to the study of Lichtenthaler^24^. The fresh leaf of *L*. *japonica* (0.5 g) under four different salt concentrations was collected and homogenized with acetone (80%, v/v). The method was as follows: Firstly, mix leaves with 50 mL 80% acetone in the flasks and ultrasonic with 5 min. Secondly, centrifuge at 8050 g for 10 min. Then absorbance of samples was determined at 441, 646, 652, and 663 nm using UV–Vis spectrophotometer (DENOVIX DS-11, USA). The contents of photosynthetic pigments (chlorophyll a, b, total chlorophyll, and carotenoids) were calculated according to the following formula:

$$ \begin{aligned} {\text{Chlorophyll}}\,{\text{a}}\left( {{\text{mg}}/{\text{g}}\,{\text{FW}}} \right) & = \left( {12.21 \times {\text{E}}663 - 2.81 \times {\text{E}}649} \right) \times \left( {{\text{V}}/1000 \times {\text{M}}} \right); \\ {\text{Chlorophyll}}\,{\text{b}}\left( {{\text{mg}}/{\text{g}}\,{\text{FW}}} \right) & = \left( {20.13 \times {\text{E}}646 - 5.03 \times {\text{E}}663} \right) \times \left( {{\text{V}}/1000 \times {\text{M}}} \right); \\ {\text{Total chlorophyll }}\left( {{\text{mg}}/{\text{g}}\,{\text{FW}}} \right) & = \left( {27.8 \times {\text{E}}652} \right) \times \left( {{\text{V}}/1000 \times {\text{M}}} \right); \\ {\text{Carotenoids }}\left( {{\text{mg}}/{\text{g}}\,{\text{FW}}} \right) & = \left[ {\left( {1000 \times {\text{E}}441} \right){-}\left( {3.27 \times {\text{Chlorophyll}}\,{\text{a}}} \right){-}\left( {104 \times {\text{Chlorophyll}}\,{\text{b}}} \right)} \right] \times [\left( {{\text{V}}/1000 \times \left( {{\text{M}} \times 229} \right)} \right] \\ \end{aligned} $$

### Proline, soluble proteins, total AA, and MDA assay

Fresh leaf samples (0.1 g) were powdered with a 1 mL sulfosalicylic acid dihydrate (SA) and transferred to a tube for 10 min heating in the boiling water bath. Afterward, centrifuged at 10,000 g for 10 min, then the supernatant was collected to detect proline concentrations at 520 nm using UV–Vis spectrophotometer (DENOVIX DS-11, USA)^25,26^. Dilute 1 mg standard substance to 15, 10, 8, 6, 4, 2, 1, 0 μg/ml, respectively. Then establishing a standard curve according to the absorption value and concentration of the standard substance.

Fresh leaf samples (0.5 g) were homogenized and extracted by 4.5 mL PBS (0.1 mol/L, PH: 7.4) in an ice-water bath. After centrifugation, the supernatant, 563 μg/mL protein standard solution, and blank solution (double distilled water) were detected using a UV–Vis spectrophotometer (DENOVIX DS-11, USA) at 562 nm, which could display the concentration of protein^27^.

Fresh leaf tissues (0.5 g) were homogenized and 4.5 mL normal saline (NS) was added, then centrifuged at 685 g for 10 min, and then took the supernatant to be measured. 1 mL supernatant mixed with 2 mL amino acid reaction solution and 1 mL amino acid chromogenic agent, and the mixture was centrifuged at 685 g for 10 min, the supernatant was then taken to measure the absorption value at 650 nm using UV–Vis spectrophotometer (DENOVIX DS-11, USA)^25^.

The content of MDA was measured by Thibabituric Acid (TBA) method^28^, which represents lipid peroxidation. 0.5 g fresh leaf tissues were grounded with 4.5 mL TBA, and centrifuged at 685 g for 10 min; 2 mL aliquot of the supernatant was mixed with 0.5% TBA, and then heated in boiling water for 40 min, quickly cooled with ice-bath. After centrifugation at 685 g for 10 min, the supernatant was prepared for the assay of MDA and the absorbance at 532 nm was recorded.

### Enzyme extraction and activities assay

The fresh leaf of *L*. *japonica* under different salt stresses were collected for determining the antioxidant activity. The leaves (0.5 g) were homogenized with 4.5 mL PBS in an ice water bath and centrifuged at 350 g (catalase, CAT), 685 g (superoxide dismutase, SOD and peroxidase, POD), and 5595 g (ascorbate peroxidase, APX) for 10 min, respectively. The supernatant was used to examine the activities of the enzyme.

The activity of SOD was assayed at 550 nm using the hydroxylamine method; the activity of POD was determined at 420 nm according to the colorimetric method^25,26^; the activity of CAT was measured at 405 nm based on the ammonium molybdate method, and the activity of APX was determined at 290 nm. All of the experiments were carried out by assay kits. The amount of 200 μL reaction solution was measured by multi-mode microplate reader using UV–Vis spectrophotometer (DENOVIX DS-11, USA).

### Chemicals and reagents

The purity of 47 standard compounds was more than 98%; the structures and source of 47 standard compounds were shown in Fig. S2 and Table S1; All of the chemicals and reagents were reported in our previous study^29,30^.

### Preparation of sample solutions

Fresh flower buds were harvested on the 20th, 25th, and 35th day, respectively, and then placed under natural dry conditions. All dried flower buds were powdered and sieved through 50 mesh. After accurately weighed powder samples (1.0 g) were extracted using ultra-sonication with 40 mL 70% methanol for 45 min, the loss of solvents was compensated with 70% methanol and mixed well. Then centrifuged at 8050 g for 10 min and filtered 0.22 nm membrane filters. The supernatants were stored in a sample bottle at 4 ℃ before UFLC-QTRAP-MS/MS analysis.

### UFLC-QTRAP-MS/MS instrumentation and conditions

The mobile phase of AB Sciex QTRAP-5500 UFLC-MS/MS spectrometry consisted of 0.2% aqueous formic acid (A) and acetonitrile with 0.2% formic acid (B). The gradient elution was as follows: 0–5 min: 2% B; 5–10 min: 2–13% B; 10–12 min: 13% B; 12–17 min: 13–25% B; 17–25 min: 25–33% B; 25–27 min: 33–35% B; 27–29 min: 35–50% B; 29–31 min: 50–95% B. The flow rate was 0.8 mL/min. Electron spray ionization (ESI) source operates in both ion modes using the multiple reaction monitoring (MRM) transition acquiring the spectra data. Except for lonicerin, kaempferol‐3‐O-rutinoside can be discriminated from different ion modes; In the same ion mode, isomers with the same ion pairs, therefore, the standard substances of isomer were sequentially injected into QTRAP‐MS/MS based on the accurate retention time to identification and quantification. Other conditions are consistent with our previous report^29^.

### Method validation and sample determination

The standard solution containing 47 compounds was prepared and the calibration curves were constructed using serially diluted standards. The LODs and LOQs of compounds were defined as signal-to-noise (S/N) ratios of 3:1 and 10:1, respectively. Method precision was calculated as the RSD of compound concentrations determined by inter-day precision. Moreover, repeatability was achieved by evaluating the closeness of a set of analytical results, which were obtained for six different replicate samples from the same sample and expressed in terms of the RSD for those measurements. The stability was measured by analyzing the variations at 0, 2, 4, 8, 12, and 24 h, respectively. At last, the recovery test was performed to check the accuracy of the method. The quantitative determination of the metabolites of LJF under salt stress was performed using the optimal condition by UFLC-QTRAP-MS/MS.

### Data processing

The data of UFLC-MS/MS were acquired and analyzed by Analyst 1.6.6 software (https://sciex.com/content/SCIEX/na/us/en/products/software/analyst-software.html). The mean values and standard deviation were calculated from the measurements of five replicates. The PLS-DA was made by SIMCA-P 13.0 (https://www.sartorius.com/en/products/process-analytical-technology/data-analytics-software/mvda-software/simca). The heat map was made by the software of Heml 1.0 Heatmap Illustrator (http://www1.heatmapper.ca/expression/). One-way ANOVA followed by the Student–Newman–Keuls test was used to compare the means with the significance level by IBM SPSS Statistics 22 (https://www.ibm.com/analytics/spss-statistics-software). Pearson Correlation Coefficient was also conducted using IBM SPSS Statistics 22 (https://www.ibm.com/analytics/spss-statistics-software), and then the difference was considered significant at *p* < 0.05. The other data were calculated by Origin Pro 9 (https://www.originlab.com/index.aspx?go=Support&pid=2051).

## Results

### Changes in morphology under salt stress

The growth parameters were shown in Table 1 and phenotype profiles of representative *L*. *japonica* presented in Fig. S3. Both leaf diameter and plant height decreased with the increasing salinity. Although average flower buds length and fresh/dry weight per 100 flower buds under high salt stress were the lowest compared with the control group, they all increased at low salt stress, indicating that salt stress had a remarkable effect on the growth parameters of *L*. *japonica*. According to the description about the grade of salt stress injury to plants, low salt stress led to the minimum damage.

**Table 1:** Changes in growth parameters of *L*. *japonica* under different levels of salt stress (the means ± SD, *n* = 3).

Treatment (mM NaCl)	Plant Height (cm)	Leaf diameter (cm)	Flower buds length (cm)	Fresh weight of per 100 flower buds (g)	Dry weight of per 100 flower buds (g)	Degree of salt stress injury
0	144.5 ± 4.57 a	6.5 ± 1.09 a	5.3 ± 0.82 a	15.2 ± 0.37 a	9.86 ± 0.30 a	0
100	142.0 ± 2.50 a	6.4 ± 0.39 a	5.6 ± 0.54 a	16.4 ± 1.03 a	10.16 ± 0.35 a	1
200	124.0 ± 2.94 b	6.0 ± 0.25 b	4.1 ± 0.42 b	13.6 ± 0.89 b	8.70 ± 0.46 b	3
300	103.0 ± 3.21 c	5.9 ± 0.10 b	3.8 ± 0.14 b	11.8 ± 1.05 c	7.91 ± 0.17 b	4

### Changes in photosynthetic pigments under salt stress

**Figure 1:**
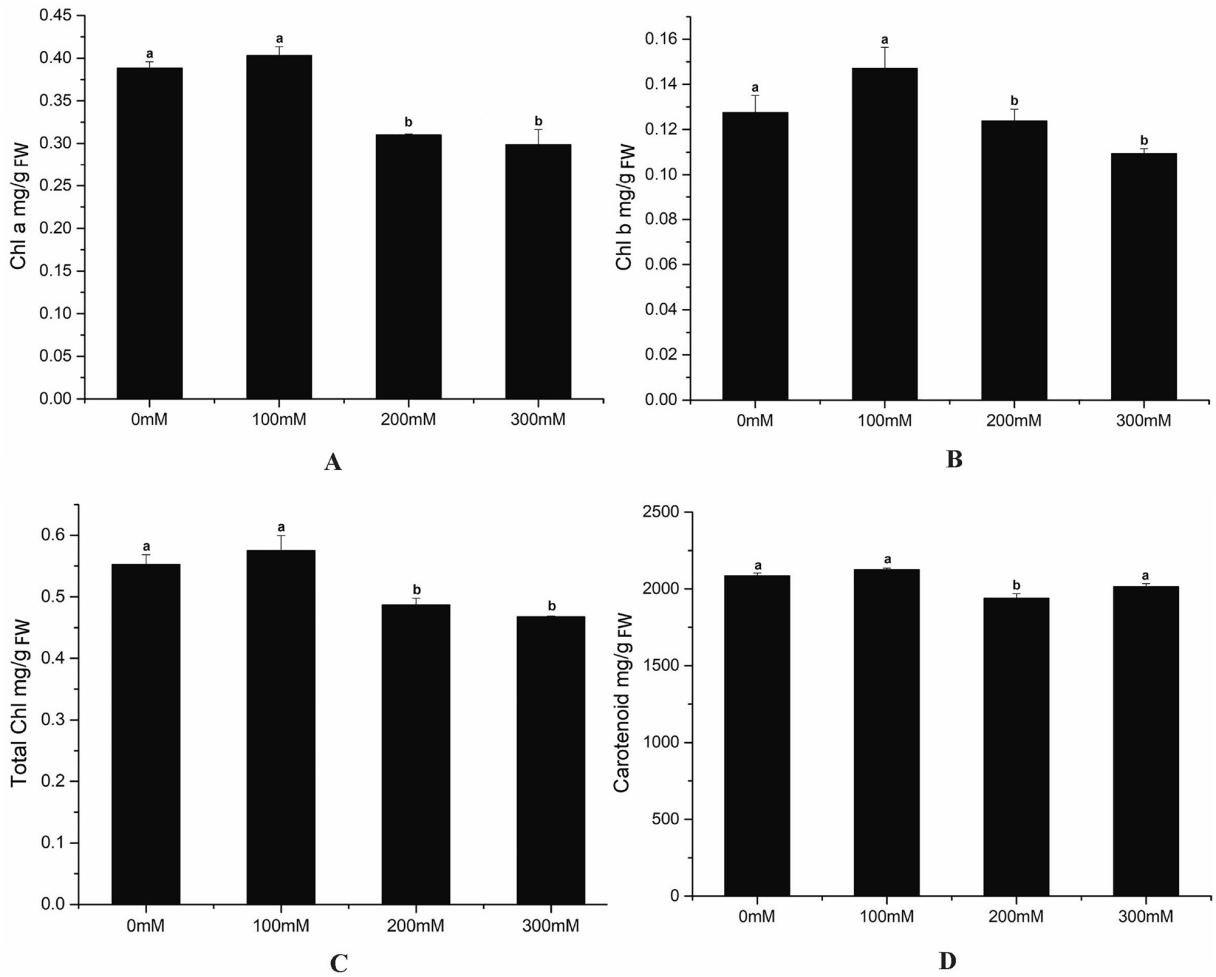
Changes in Photosynthetic Pigments contents under different NaCl concentrations. Chlorophyll a (**A**), Chlorophyll b (**B**), Total chlorophyll (**C**), Carotenoid (**D**) (The bars were standard deviation (SD) in figure).

As shown in Fig. 1, the content of photosynthetic pigments increased firstly and then decreased except carotenoid; but the increments were insignificant. Among them, low salt stress occupied the highest values, indicating that this treatment significantly increased chlorophyll a, chlorophyll b, total chlorophyll, and carotenoids concentration.

### Changes in osmolytes, and lipid peroxidation under salt stress

**Figure 2:**
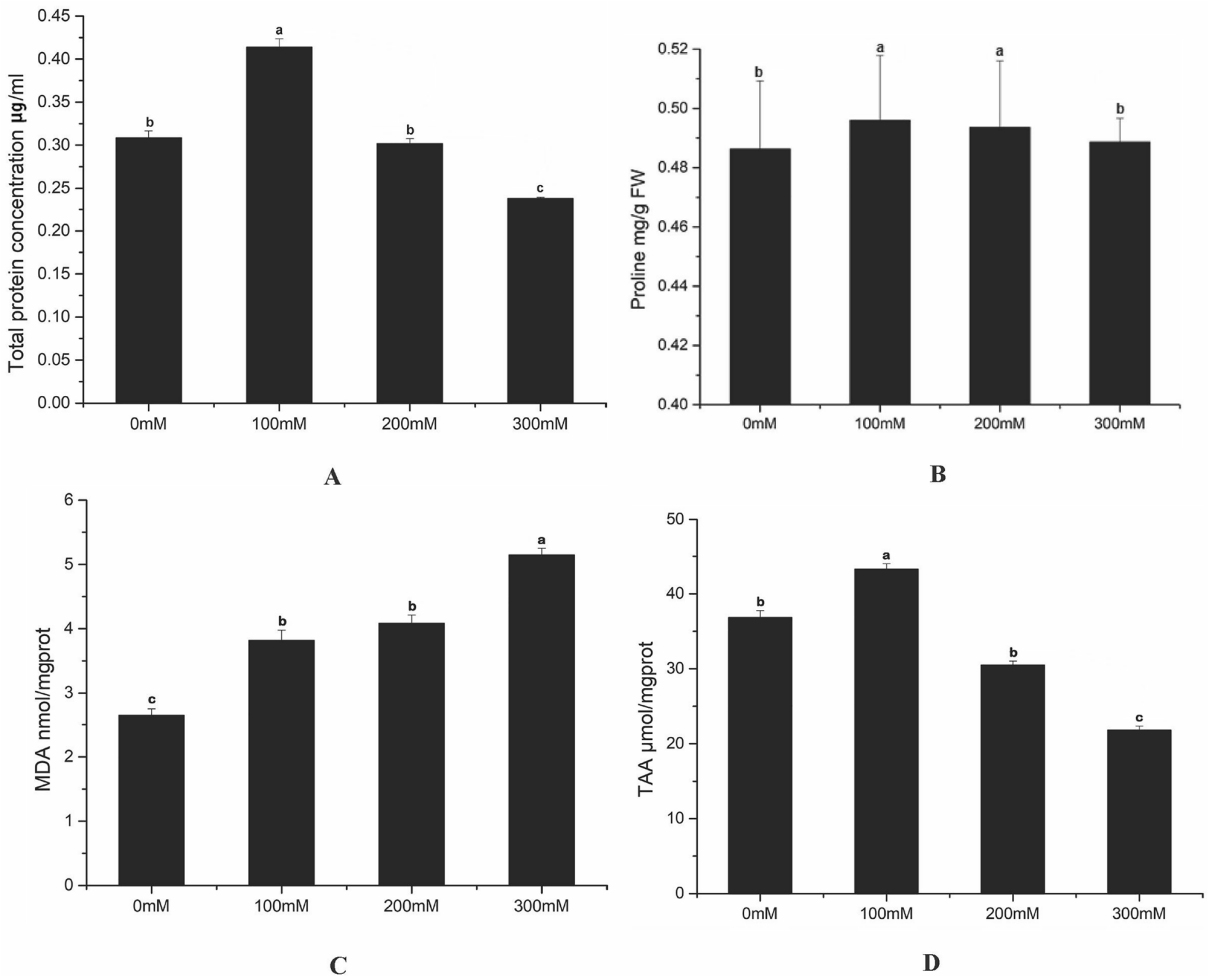
Changes in soluble proteins (**A**), proline (**B**), MDA (**C**), and TAA (**D**) under different concentrations of NaCl (The bars were standard deviation (SD) in figure).

In the experiment, we identified osmolytes including soluble sugars, soluble proteins, and proline; both soluble proteins and proline had higher values under low salt stress (Fig. 2A,B). The contents of proline also increased compared with the control group. This increase occurred in the early stage of salt stress with low salinity, while with the lasting salinity increase, the proline concentration showed the opposite trend. It is well acknowledged that the content of MDA was quantified as the estimate of membrane lipid peroxidation, which represented the degree of cell membrane damage. As shown in Fig. 2C, MDA continued to accumulate when salinity increases from 100 to 300 mM. This phenomenon indicated that *L*. *japonica* tolerated salinity, and the oxidative damage peaked under 300 mM salt stress. Amino acids can provide the material basis for normal growth, metabolism, and life support. It can be seen from Fig. 2D that the content of total amino acids reached the maximum 43.31 μmol/mgprot, and then almost straight down.

### Changes in antioxidant enzyme activity under salt stress

**Figure 3:**
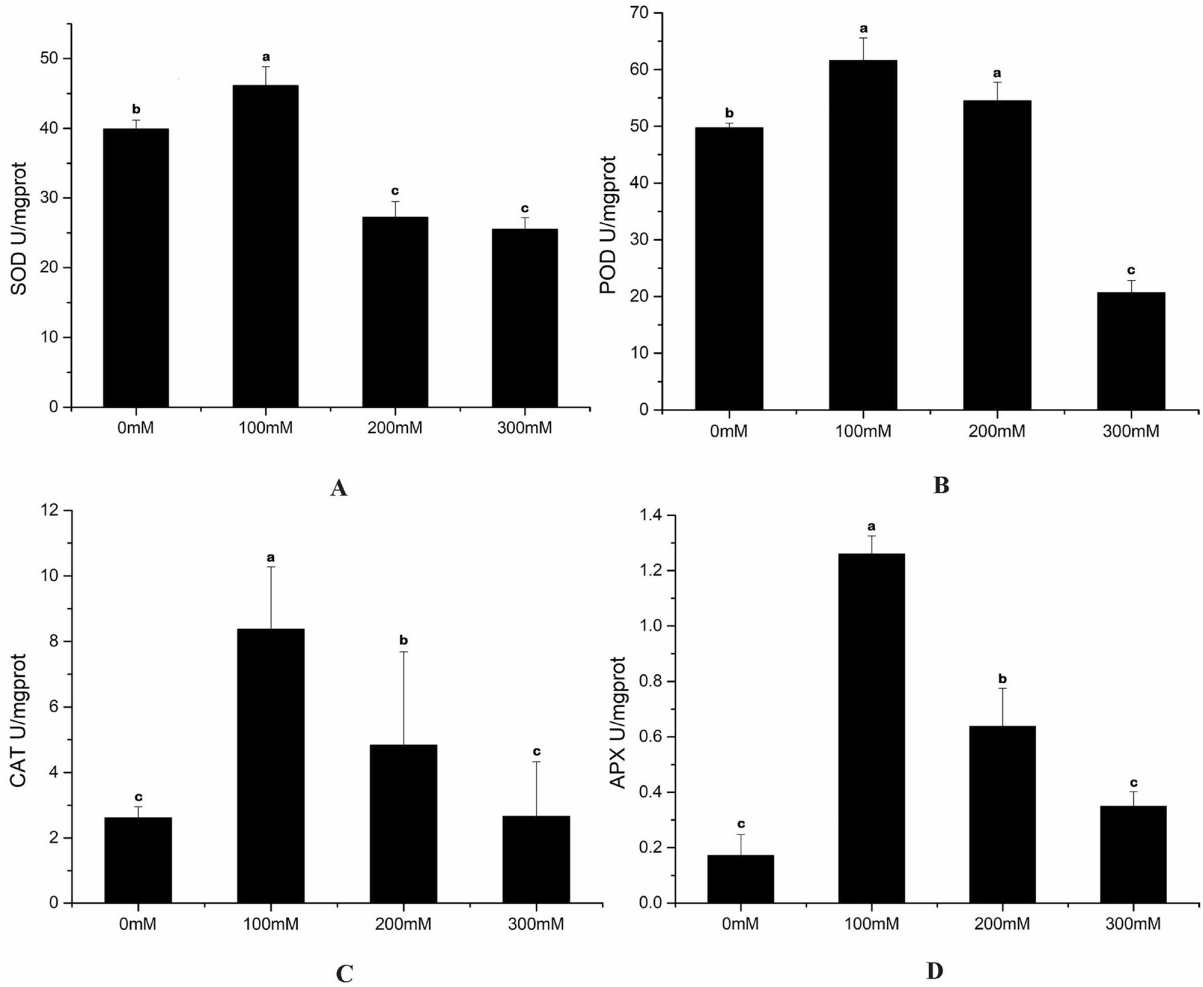
Changes in superoxide dismutase (SOD, **A**), peroxidase (POD, **B**), catalase (CAT, **C**), ascorbate peroxidase (APX, **D**) activity under different concentrations of NaCl (The bars were standard deviation (SD) in figure).

Results from Fig. 3 showed that the activity of POD, SOD, CAT, and APX varied with different concentrations of NaCl which peaked at 100 mM NaCl treatment. POD activity peaked at 61.68 U/mg, but suddenly declined by 2.98-fold from 100 to 300 mM. CAT activity induced by 3.2-fold at 100 mM compared with controls. And APX activity peaked at 1.26 U/mg firstly, then declined by 7.38-fold compared with controls which accompanied with increasing salinity from 100 to 300 mM.

### Optimization of UFLC-QTRAP-MS/MS conditions

**Figure 4:**
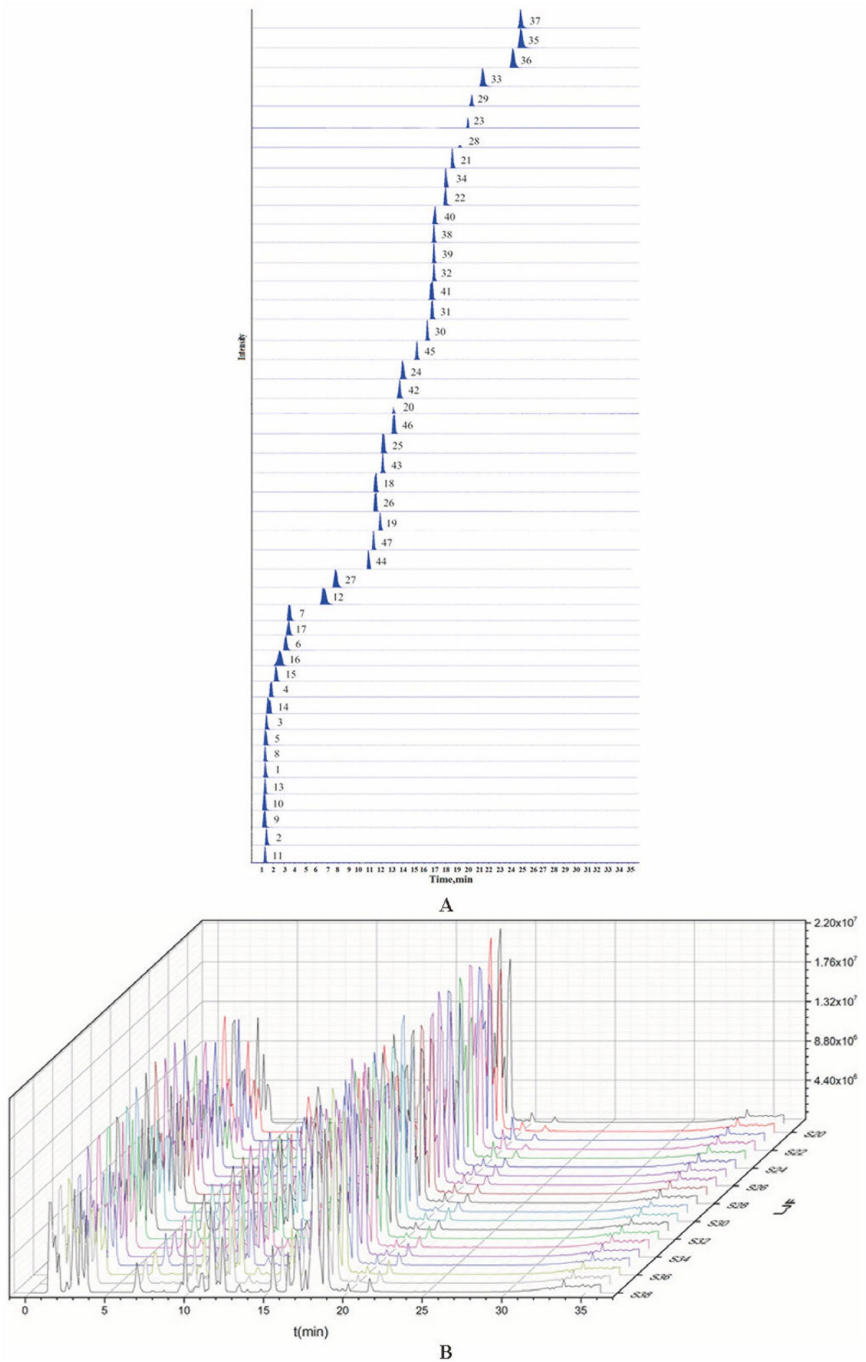
Multiple Reaction Monitoring (MRM) of 47 compounds in LJF (**A**); Overlay chart of extracted ion chromatograms (EICs) of LJF (**B**).

The optimum UFLC-QTRAP-MS/MS conditions were modified based on our previous work^29^. The most abundant and specific fragment ions were selected as MRM transition from MS/MS and the detailed information of 47 compounds was summarized in (Table S2). The representative extract ion chromatograms of 47 active constituents in the MRM mode were shown in Fig. 4A; the total ion chromatograms (TIC) were presented in Fig. 4B.

### Method validation

The detailed information of the method validation for UFLC-QTRAP-MS/MS was listed in Table 2. All of the compounds showed good linearity with *r* > 0.9990. The LODs and LOQs of 47 constituents ranged from 0.002–167.42, 0.008–552.48 ng/mL, respectively. The RSD values of precision, repeatability, stability of 47 constituents were less than 5%. The recoveries were measured in the range of 94.2–105.2%.

**Table 2:** Regression equations, limit of detection (LOD), limit of quantification (LOQ), precisions, repeatabilities, stabilities, and recoveries for 47 bioactive components of Lonicerae Japonicae Flos.

Number	Name	CAS no	Formula	Regression equation	*r*	Linear range (ng/mL)	LOD(ng/ml)	LOQ(ng/ml)	Precision (RSD%; *n* = 6)	Repeatability	Stability	Recovery	
										(RSD%; *n* = 6)	(RSD%; *n* = 6;)	Mean	RSD%
1	L-Alanine	56-41-7	C_3_H_7_NO_2_	*y* = 4.36E^4^ + 1.1E3*x*	0.9995	2.06–25,800	0.052	0.194	1.65	2.47	4.32	100.08	4.56
2	L-Serine	56-45-1	C_3_H_7_NO_3_	*y* = 6.13E^4^ + 3.07E^3^*x*	0.9996	1.04–12,550	0.100	0.347	1.87	3.73	1.72	99.56	1.98
3	L-Proline	147-85-3	C_5_H_9_NO_2_	*y* = 2.28E^5^ + 1.17E^4^*x*	0.9996	5.25–13,125	0.053	0.210	1.80	4.23	3.63	101.36	2.39
4	L-Valine	72-18-4	C_5_H_11_NO_2_	*y* = 1.28E^5^ + 4.95E^3^*x*	0.9999	18.46–46,615	0.037	0.133	3.98	3.74	4.26	98.55	3.86
5	L-Threonine	72-19-5	C_4_H_9_NO_3_	*y* = 8.23E^3^ + 384*x*	0.9992	4.024–40,240	0.008	0.029	3.00	4.16	4.29	96.76	4.18
6	L-Isoleucine	73-32-5	C_6_H_13_NO_2_	*y* = 2.97E^5^ + 1.5E^4^*x*	0.999	1.22–15,210	0.012	0.039	3.74	3.77	4.11	100.09	2.15
7	L-Leucine	61-90-5	C_6_H_13_NO_2_	*y* = 3.23E^5^ + 2.69E^4^*x*	0.9995	1.03–12,875	0.010	0.033	4.50	3.98	0.37	102.97	2.06
8	L-Aspartic acid	56-84-8	C_4_H_7_NO_4_	*y* = 1.05E^5^ + 1.8E^3^*x*	0.9998	5.03–25,152	0.050	0.161	3.19	2.94	3.05	99.27	2.96
9	L-Glutamate	138-18-1	C_5_H_7_NO_4_	*y* = 4.8E^4^ + 554*x*	0.9995	10.32–129,030	0.103	0.310	4.26	4.81	3.05	96.93	3.96
10	L-Lysine	56-87-1	C_6_H_14_N_2_O_2_	*y* = 7.67E^4^ + 773*x*	0.9991	9.11–113,960	0.091	0.274	3.26	3.65	2.62	95.67	3.73
11	L-Histidine	71-00-1	C_6_H_9_N_3_O_2_	*y* = 3.48E^3^−6.58E^3^*x*	0.9999	2.12–8,512	0.004	0.015	0.47	2.94	2.34	105.2	4.64
12	L-Phenylalanine	63-91-2	C_9_H_11_NO_2_	*y* = 3.21E^5^ + 1.94E^4^*x*	0.9992	1.09–13,650	0.011	0.033	1.16	3.01	1.87	97.56	3.06
13	L-Arginine	74-79-3	C_6_H_14_N_4_O_2_	*y* = 2.55E^4^ + 1.49E^3^*x*	0.9997	6.87–34,340	0.172	0.515	2.82	1.32	9.83	100.17	1.98
14	Cytidine	65-46-3	C_9_H_13_N_3_O_5_	*y* = 2.51E^3^ + 7.17E^3^*x*	1	0.2224–11,120	0.056	0.167	1.84	2.61	3.41	94.2	3.9
15	Uridine	58-96-8	C_9_H_12_N_2_O_6_	*y* = 2.15E^3^ + 1.44E^3^*x*	0.9990	1.72–21,520	0.043	0.129	2.57	4.60	3.41	100.95	2.55
16	Adenosine	58-61-7	C_10_H_13_N_5_O_4_	*y* = 13.53E^4^ + .35E^4^*x*	0.9990	0.21–2,580	0.002	0.008	4.10	2.56	1.99	97.76	3.7
17	Inosine	58-63-9	C_10_H_12_N_4_O_5_	*y* = 1.32E^5^ + 2.78E^4^*x*	0.9997	2.47–6,180	0.124	0.492	3.55	1.35	3.13	101.58	4.12
18	Chlorogenic acid	327-97-9	C_16_H_18_O_9_	*y* = 5.46E^5^ + 5.05E^3^*x*	0.9992	24.48–10,100	1.224	4.039	3.30	2.65	4.87	99.77	1.22
19	Neochlorogenic acid	906-33-2	C_16_H_18_O_9_	*y* = 4.17E^5^ + 243*x*	0.9995	837.08–88,375	167.418	552.478	4.82	4.34	4.02	96.7	3.97
20	Cryptochlorogenic acid	905-99-7	C_16_H_18_O_9_	*y* = 6.54E^5^ + 1.17E^4^*x*	0.9993	527.5–41,800	105.500	316.500	1.70	2.33	3.67	100.66	1.62
21	Isochlorogenic A	2450-53-5	C_25_H_24_O_12_	*y* = 8.34E^5^ + 5.81E^3^*x*	0.9990	31.64–92,250	0.633	2.278	2.02	1.87	3.23	98.7	2.65
22	Isochlorogenic B	14,534-61-3	C_25_H_24_O_12_	*y* = 5.95E^5^ + 4.88E^3^*x*	0.9998	36.96–227,900	7.392	24.394	5.00	2.09	2.36	100.67	3.65
23	Isochlorogenic C	57,378-72-0	C_25_H_24_O_12_	*y* = 2.84E^5^ + 8.24E^4^*x*	0.9996	3.21–59,400	0.032	0.125	1.31	3.48	1.83	99.4	2.05
24	1,3-O-dicaffeoylquinic acid	19,870-46-3	C_25_H_24_O_12_	*y* = 5.21E^5^ + 2.03E^4^*x*	0.9994	119.9–24,480	23.980	71.940	1.57	2.26	1.65	100.95	3.46
25	Caffeic acid	331-39-5	C_9_H_8_O_4_	*y* = 9.74E^4^ + 5.23E^3^*x*	0.9996	110.5–24,840	22.100	77.350	1.02	1.61	2.35	101.96	2.12
26	Quinic acid	77-95-2	C_7_H_12_O_6_	*y* = 1.06E^5^ + 281*x*	0.9996	11.05–24,192	0.685	2.192	3.90	2.64	2.35	100.92	2.28
27	Protocatechuic acid	99-50-3	C_7_H_6_O_4_	*y* = 5.71E^4^ + 1.88E^4^*x*	0.9991	6.85–25,665	1.100	3.630	1.95	2.35	3.39	99.65	2.8
28	Ferulic acid	1135-24-6	C_10_H_10_O_4_	*y* = 2.33E^3^−101*x*	0.9999	3.65–16,150	0.866	2.858	3.62	3.05	2.47	101.22	3.68
29	4,5-O-dicaffeoylquinic acid methyl ester	114,637-83-1	C_26_H_26_O_12_	*y* = 9.8E^3^ + 4.59E^3^*x*	0.9994	0.36–96,390	0.018	0.072	2.04	3.11	4.47	97.98	4.48
30	Rutin	153-18-4	C_27_H_30_O_16_	*y* = 1.31E^5^ + 2.4E^3^*x*	0.9997	4.97–9,432	0.497	1.639	2.42	3.50	4.92	98.62	4.01
31	Hyperoside	482-36-0	C_21_H_20_O_12_	*y* = 9.41E^4^ + 1.11E^4^x	0.9999	14.21–16,950	0.711	2.132	0.39	1.94	2.41	99.85	3.82
32	Luteoloside	5373-11-5	C_21_H_20_O_11_	*y* = 1.02E^5^ + 2.11E^3^*x*	0.9994	0.71–7,537	0.081	0.322	2.11	2.02	4.36	99.56	2.95
33	Luteolin	491-70-3	C_15_H_10_O_6_	*y* = 3.21E^5^ + 5.7E^3^*x*	0.9994	25.67–8,220	1.283	5.116	1.68	2.25	3.43	100.89	4.25
34	Rhoifolin	17,306-46-6	C_27_H_30_O_14_	*y* = 1.95E^3^−6.96E^3^*x*	0.9995	0.17–17,250	0.007	0.028	3.90	4.65	2.89	99.92	3.12
35	Diosmetin	520-34-3	C_16_H_12_O_6_	*y* = 2.19E^4^ + 1.33E^4^*x*	0.9998	0.27–14,210	0.003	0.012	1.01	3.13	2.17	100.76	3.05
36	Apigenin	520-36-5	C_15_H_10_O_5_	*y* = 1.21E^4^ + 1.4E^4^*x*	0.9999	0.02–18,000	0.002	0.008	0.70	2.79	3.54	103.29	3.58
37	Kaempferol	520-18-3	C_15_H_10_O_6_	*y* = 2.81E^3^ + 527*x*	0.9992	0.17–86,620	0.034	0.119	0.86	4.40	1.66	101.27	3.09
38	Astragalin	480-10-4	C_21_H_20_O_11_	*y* = 3.97E^4^ + 5.71E^3^*x*	0.9995	0.19–66,00	0.038	0.132	0.72	3.57	5.48	100.87	3.77
39	Lonicerin	25,694-72-8	C_27_H_30_O_15_	*y* = 1.91E^5^ + 992*x*	0.9998	9.64–342,500	9.639	28.917	2.44	0.65	4.57	100.77	2.08
40	Kaempferol-3-O-rutinoside	17,650-84-9	C_27_H_30_O_15_	*y* = 3.74E^5^ + 3.48E^4^*x*	0.9998	2.24–5,610	0.022	0.074	1.50	4.72	3.12	99.68	3.16
41	Isoquercitrin	482-35-9	C_21_H_20_O_12_	*y* = 2.74E^5^ + 7.34E^3^*x*	0.9996	3.23–27,625	0.808	2.423	0.78	2.06	2.25	99.58	2.35
42	Sweroside	14,215-86-2	C_16_H_22_O_9_	*y* = 4.83E^3^ + 79.8*x*	0.9993	4.75–5,995	0.475	1.426	2.39	2.03	2.55	101.78	2.95
43	Secologanic acid	60,077-46-5	C_16_H_22_O_10_	*y* = 2.01E^5^ + 635*x*	0.9992	18.23–18,480	1.823	6.199	1.76	3.58	3.11	99.82	4.02
44	Loganin acid	22,255-40-9	C_16_H_24_O_10_	*y* = 1.27E^5^ + 1.54E^3^*x*	0.9997	0.74–15,820	0.074	0.286	4.51	4.55	4.47	103.66	3.79
45	Loganin	18,524-94-2	C_17_H_26_O_10_	*y* = 1.45E^3^ + 12.8*x*	0.9990	2.09–1,605	0.084	0.324	1.28	3.68	4.95	98.78	3.14
46	Secoxyloganin	58,822-47-2	C_17_H_24_O_11_	*y* = 1.04E^5^ + 1.46E^3^*x*	0.9997	0.71–418,544	0.071	0.283	1.16	3.63	3.46	100.02	2.76
47	Morroniside	25,406-64-8	C_17_H_26_O_11_	*y* = 783 + 324*x*	0.9996	0.20–105,50	0.010	0.039	3.43	4.00	3.29	100.75	4.19

### The accumulation of bioactive constituents of all groups in LJF

**Figure 5:**
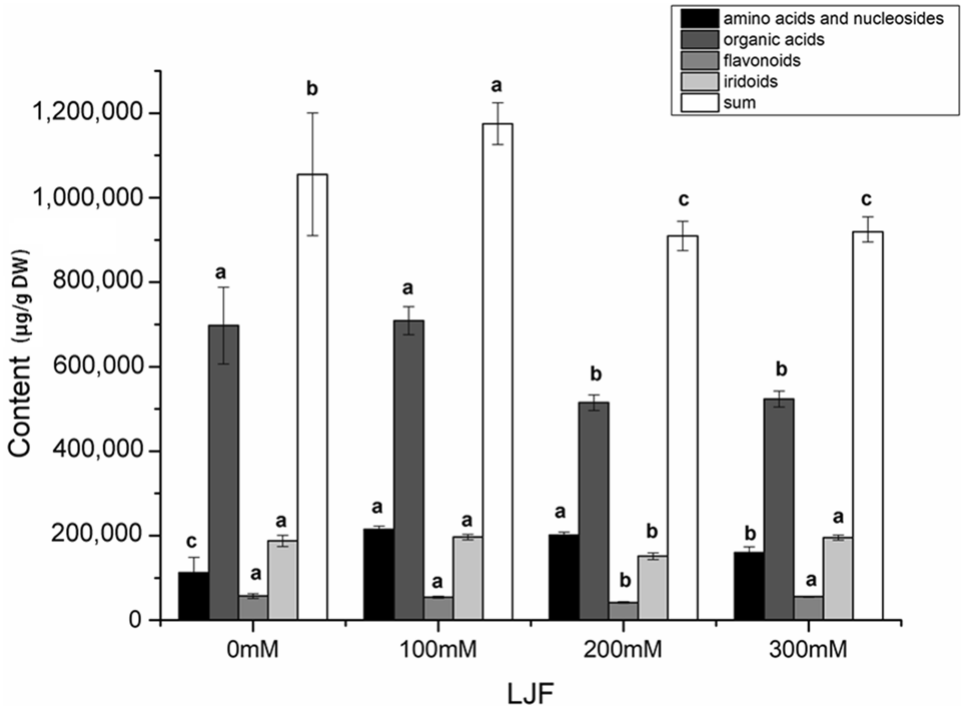
The accumulation of primary and secondary metabolites under salt stress in LJF. Bars are express as the mean ± SD (*n* = 5), the letters of bars indicate significant differences at *p* < 0.05.

The detailed changes of bioactive constituents during 35 days in LJF were obtained. According to Table S3 and Fig. 5, the variation trends of amino acids, nucleosides, organic acids, and flavonoids increased and then decreased. Conversely, iridoids concentration peaked under 300 mM, such as loganin secoxyloganin and morroniside increased 3.98, 3.10, and 9-folds compared to controls, respectively. The contents of chlorogenic acid and its derivatives were also enhanced under salt stress, including isochlorogenic A and B were elevated 2.32 and 2.31-folds, respectively. Among them, the trends of chlorogenic acid, neochlorogenic acid, isochlorogenic A, and isochlorogenic B were accordant, which indicated that the accumulation and changes of pathway-related constituents interacted with each other. From the chemical fingerprint and the content data, the chemical fingerprint was similar, but the content of individual constituents or total contents was different under different salt stress. The contents of organic acids were the most abundant under different salt stress. And the total LJF biomass reached a peak by 1.1-fold after 35 days under low salt stress compared with controls. Altogether, we found that the environment had an obvious effect on the accumulation of bioactive constituents, the accumulation of active constituents was the highest under low salt stress.

### Dynamic accumulation of multiple bioactive constituents

**Figure 6:**
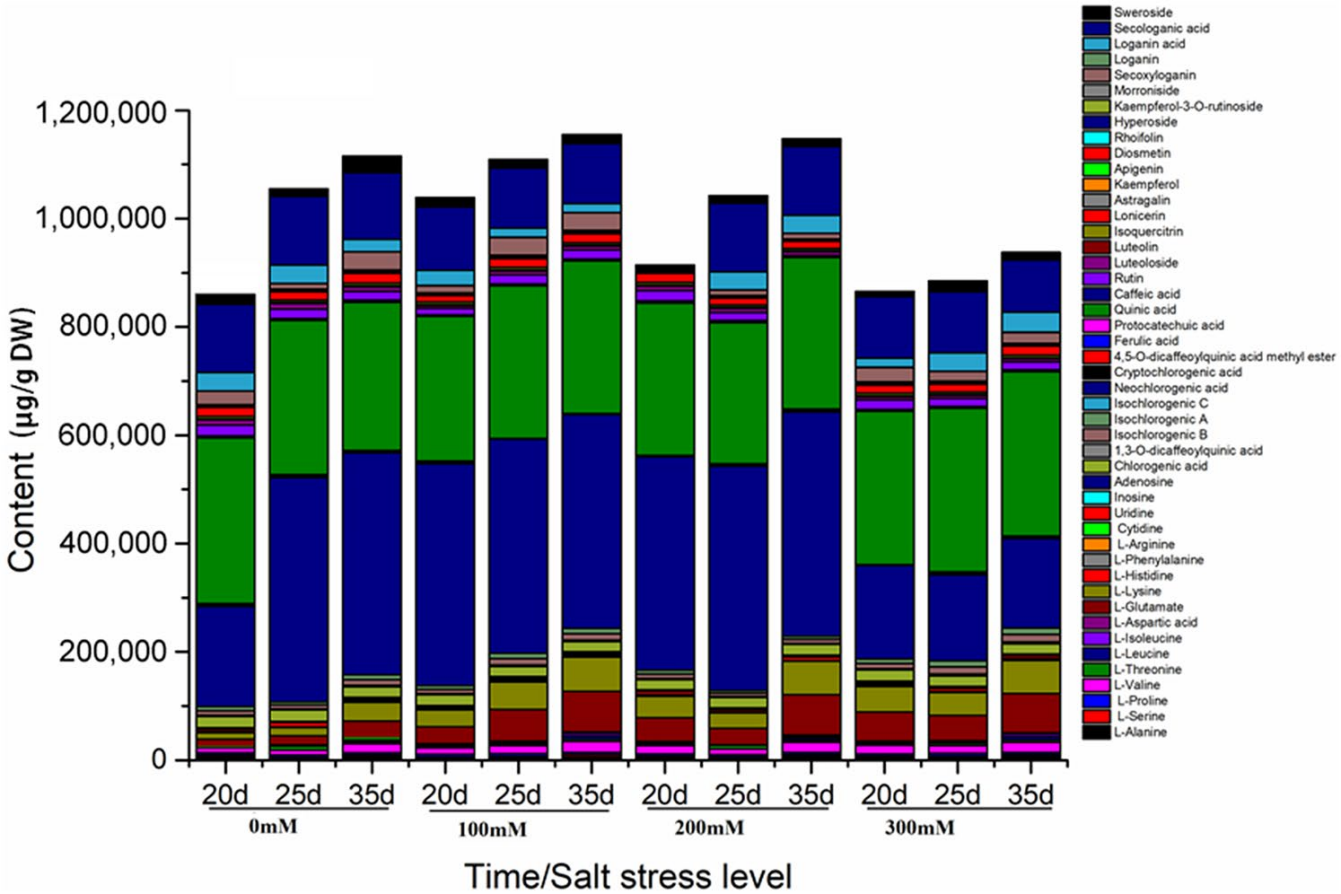
Accumulation contents of 47 compounds in 20, 25, 35 days, respectively.

As shown in Fig. 6, although the accumulation of bioactive constituents under four salinity levels increased with time (20, 25, and 35 days, respectively), the dynamic accumulation of these constituents still exhibited some differences. As the accumulation of bioactive constituents peaked at 100 mM, we supposed that low salt stress might be the optimum condition for accumulating bioactive constituents of LJF, which represented the highest medicinal. As one of the indicator constituents to evaluate the quality of LJF, the dynamic accumulation and changing trend of chlorogenic acid was firstly increased and then decreased in the whole process. And the incremental accumulation became greater with time prolonging under 100 mM treatment. This also suggested that the environment has a great impact on the accumulation of active constituents and the different accumulation patterns were presented in the four different salt stress levels. In the long run, low salt stress (100 mM) instead of high salt stress (300 mM) was beneficial for biomass accumulation and growth of LJF.

**Figure 7:**
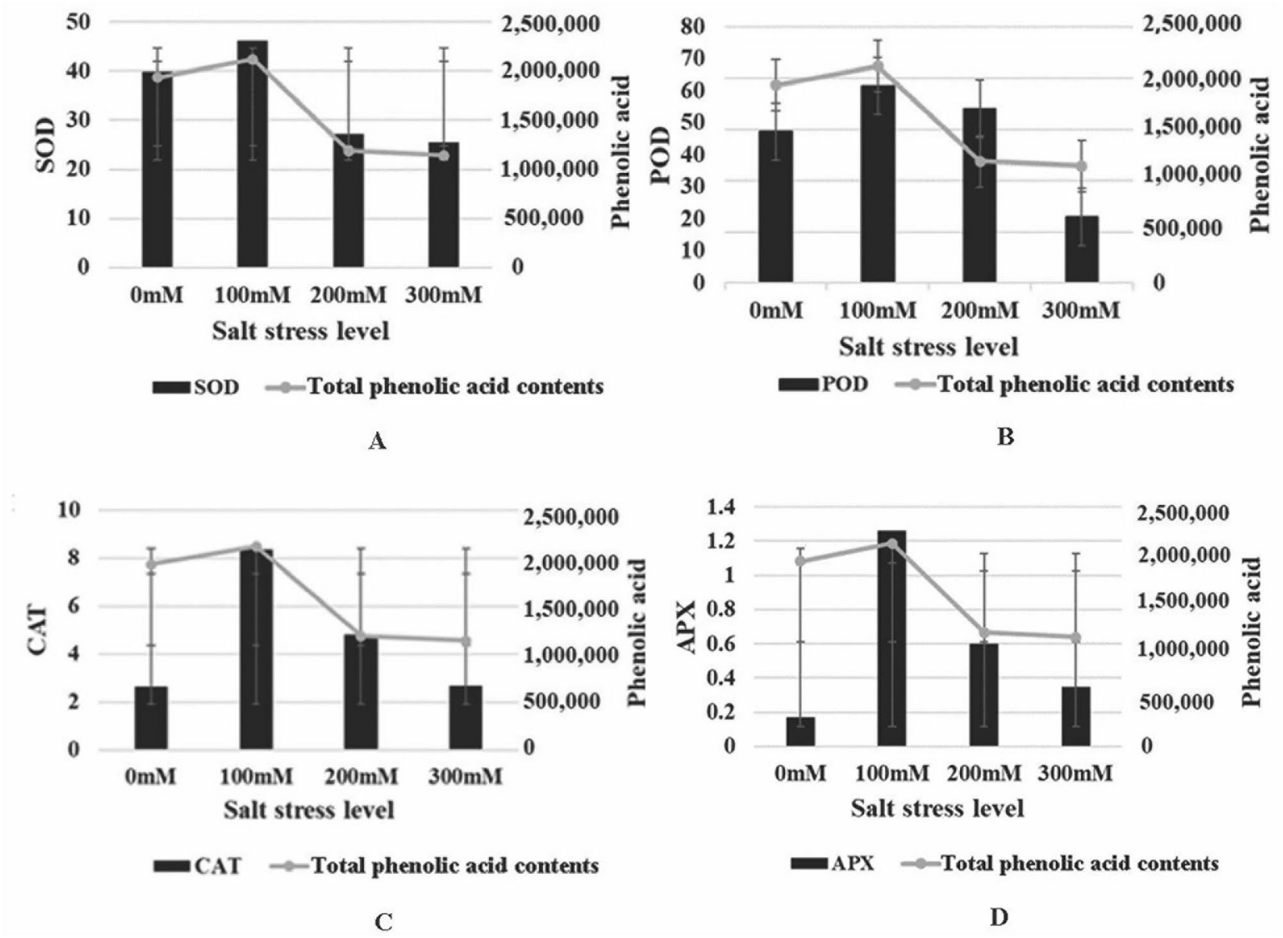
The correlation between antioxidant enzyme activity (superoxide dismutase (SOD, **A**), peroxidase (POD, **B**), catalase (CAT, **C**), ascorbate peroxidase (APX, **D**)) and total phenolic acid content under salt stress.

According to the traditional viewpoint, phenolic constituents were regarded as secondary antioxidants and played a vigorous role in detoxifying ROS. In this study, we found that soil salinity considerably affected phenolic acid contents and antioxidant activity. In Fig. 7, the contents of phenolic acids were positively correlated with antioxidant enzymes activity (SOD, POD, CAT, and APX activity) under salt stress, which were decreased with the reducing phenolic acid contents from 100 to 300 mM, indicating that the phenolic acids positively correlated with oxidative damage reduction. Pearson Correlation Coefficient (r) was performed to further analyzed the correlations between phenolic acid contents and antioxidant enzymes under salt stress. the results showed that the r-value of SOD, POD, CAT, and APX activity were 0.994, 0.989, 0.532, and 0.416, respectively. Among them, the correlation between phenolic acid contents and SOD, POD activity was significant correlation at 0.01, 0.05 level, respectively.

### Multivariate statistical analysis

**Figure 8:**
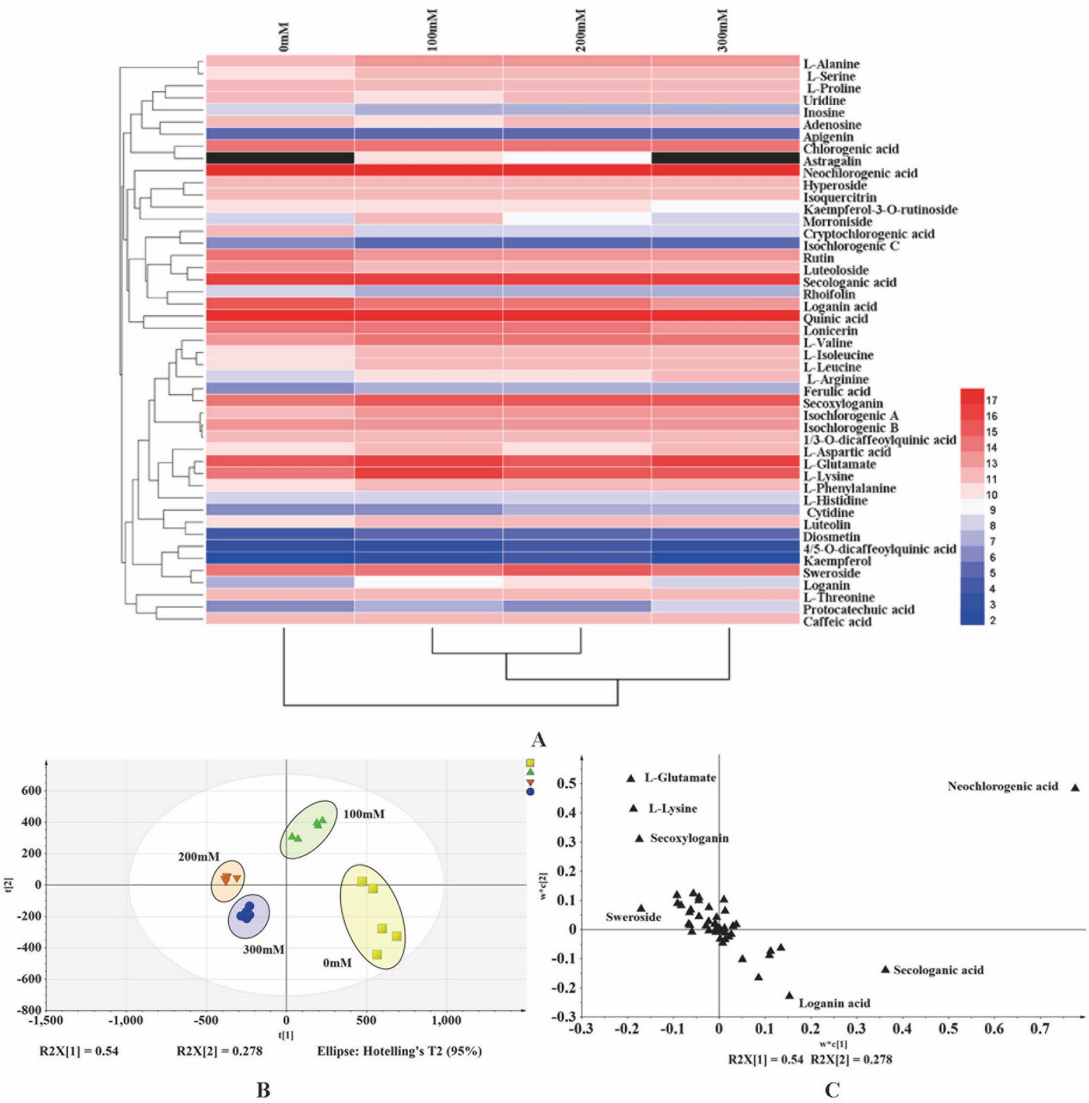
Multivariate Statistical Analysis of LJF under different salinity level. hierarchical clustering heat map (**A**), PLS-DA score plot (**B**), PLS-DA loading plot (**C**).

The hierarchical clustering heat map displayed the changes in the accumulation of 47 constituents under different salt stresses. As shown in Fig. 8A, the changes of 47 constituents in LJF indicated that the environment influenced the accumulation of bioactive constituents. And the specific cases in the control group and salt stress groups were separated. In the different salt stress groups, 100 mM and 200 mM group were firstly clustered together and then separated. Finally, the four different salt stress groups were completely separated.

The PLS-DA was performed to cluster and classify LJF under different salt stresses. Samples were divided into four groups with *R*^*2*^*X* = 0.959, *R*^*2*^*Y* = 0.974, and *Q*^*2*^ = 0.871, respectively (Fig. 8B); of which the values represented excellent fitness (*R*^*2*^*X/ R*^*2*^*Y*) and high predictive ability (*Q*^*2*^). And in the PLS-DA loading plot (Fig. 8C), L-glutamate, L-lysine, neochlorogenic acid, sweroside, loganin acid, secologanic acid, and secoxyloganin possessed great loading values of ions, enhancing to separate samples.

Due to GRA prefers to deal with complex relationship problems with multiple factors and variables like traditional Chinese medicine, it was carried out to evaluate the quality of LJF based on the contents of 47 bioactive constituents. Grey comprehensive evaluation values (*ri*) and quality ranking were listed in Table 3, it showed that the quality ranking of LJF was 100 mM at the top, followed by 0, 200, and 300 mM; and the *ri* values were 0.75, 0.52, 0.29, and 0.25, respectively. It manifested LJF could be grown in soil with 100 mM NaCl and obtained the optimum quality.

**Table 3:** Quality sequencing of the Lonicerae Japonicae Flos samples affected by NaCl.

Treatments (mM)	*ri*	Quality-ranking
0	0.52	2
100	0.75	1
200	0.29	3
300	0.25	4

## Discussion

The changes in morphology, physiology, and metabolites of medicinal plants are adaptors to adversity and provide a self-protection mechanism. Salinity has become a worldwide problem that serves as ubiquitous abiotic stress affecting the growth, quality, and yield of medicinal plants. When plants suffer from salt stress, the absorption, accumulation, and distribution of water and nutrients are affected, triggering morphological changes of plants, this is a visual change^31^. Growth parameters and biomass are the most direct indicators of salt tolerance, and then plant height and fresh weight can reflect the biomass of plants^32^. According to these issues, it is inferred that flower buds treated with 100 mM NaCl might be the maximum accumulation of biomass. The decrease at 200 and 300 mM NaCl might be due to the injury caused by osmotic stress, and ion toxicity caused by the accumulation of Na^+^ ions, which hinder absorption of K^+^ ions^33^.Therefore, we think that different salt stresses had different influences on the process of growth and development.

The content of MDA is regarded as a reliable indicator of membranes' oxidative damages and has antioxidant activity through the protection of cellular membranes and enzyme integrity^34^. In this study, the contents of MDA increase with salinity, which indicate salt aggravates oxidative damage of LJF. Proline is an important osmolyte in plants, its accumulation is a defense response to salt stress^35^. Proline not only promotes osmotic homeostasis, but also associated with the salt tolerance of LJF. This is consistent with previous results on wheat^36^, wild chicory^37^. Besides, as a protein precursor, it is the energy source of the stress recovery process^38^. Amino acids and biological enzymes are also primary metabolites of plants and play a role in maintaining osmotic balance^39^. According to the results, total amino acids content and photosynthesis/antioxidant enzyme activity peak at 100 mM salt condition, which is optimal salt concentration.

Photosynthesis is a prerequisite for keeping plant normal growth. Therefore, to study the effects of salt stress on photosynthetic parameters is of great significance for improving salt tolerance and the quality of medicinal plants. The strength of photosynthesis can be used as an indicator for judging plant growth and stress resistance^40^, and the content of Chlorophyll b directly affects the photosynthetic capacity of plants. Here, we find that chlorophyll b concentration peak at 100 mM NaCl. Compared with the control group, the contents of photosynthetic pigments are elevated under low salt stress, but reduced under high salt stress, indicating that low salt stress could boost the photosynthesis of LJF. Conversely, high salt stress inhibits^41^.

It is known that high salt concentration causes osmotic pressure, resulting in an oxidation imbalance in the plant cells; sequentially, causing damage to proteins and cell membrane integrity^42^. In order to mitigate oxidative damage, plants have developed a series of antioxidant responses, including osmotic balance, antioxidant enzymes, and metabolites. The changes in antioxidant enzymes (POD, SOD, APX, and CAT) imply that salt stress stimulates the antioxidant system. The increase of APX activity is accompanied by the increase of SOD, CAT activity, which is referred to as a common regulation for active oxygen scavenging. Also, APX is a key enzyme to protect chloroplasts and other cellular components from H_2_O_2_ and hydroxyl radicals. Our findings revealed that POD, SOD, APX, and CAT activity are induced under low salt stress, suggesting that antioxidant enzymes perform a protective effect on preventing adverse reactions. Also, the correlation between phenolic acid contents and antioxidant enzymes under salt stress is consistent with the previous research^18^; our results add further support for this conclusion.

Primary metabolites not only constitute essential nutrients for plant growth, but also protect cellular membranes integrality under salt stress^43^. In this study, the contents of serine, glutamate, and lysine are elevated, which could play a positive role in osmotic adjustments. Parts of nucleosides and their derivatives have significant physiological functions. For instance, inosine is beneficial in the treatment of rheumatic heart disease, acute, and chronic hepatitis. Nucleoside is the main component of nucleic acid. And nucleic acid is the precursor to the synthesis of RNA and DNA. In the study, the contents of nucleoside are relatively lower under salt stress, as salt could cause protein structure changes, increase cytoplasmic RNA enzyme activity, and then lead to a decrease in DNA synthesis^44^. Additionally, Li et al.^45^ discussed the important secondary metabolites and their variation in plants under salt stress in detail, which reveal salt stress improves the accumulation of various secondary metabolites possessing antioxidant activity; such as phenolics and flavonoid metabolites in this study. Phenolic cooperates with the POD to scavenge H_2_O_2_. Flavonoid metabolites clear free radicals with the help of phenolic hydroxyl groups. The phenylpropanoid pathway is the main metabolic pathway for the synthesis of phenolics and flavonoids. Chlorogenic acid and its derivatives are the main phenolic acid constituents and have important quantitative significance both in LJF and other medicinal plants. Previous studies^18,46,47^ have demonstrated that the accumulation and synthesis of chlorogenic acid and luteoloside are directly affected by the variation in hydroxycinnamoyl-Co A quinate hydroxycinamoyl transferase (HQT) and chalcone flavanone isomerase (CHI) activity. Therefore, the correlation between variations of main effective constituents (phenolic acids, flavonoids, and iridoids) contents and the activities of their associated regulatory enzymes under salt stress, which is a promising point and worthy of in-depth study. It is worth mentioning that the content of morroniside increase ninefold under 100 Mm NaCl treatment and plays a protective role in oxidative stress and myocardial injury. This inspires that even less bioactive constituent content, reprogramming related metabolic pathways through salinity induction also induces the production of these active substances, which provides new clues for improving the quality of medicinal plants. According to the traditional view, the production of secondary metabolites consume a large chunk of energy, which is evident from the plants' limited growth and lipid peroxidation under high salt^48^. Phenolic constituents are regarded as secondary antioxidants and play a vigorous role in detoxifying ROS. In our results, the accumulation of phenolic constituents is induced in low salt stress, while the accumulation is reduced under high salt stress. And Pearson Correlation Coefficient is performed to further verified the correlations. The result show that between phenolic acid contents and SOD, POD activity is significant correlation at 0.01, 0.05 level, respectively. Our findings are in accordance with that the low salinity is beneficial to the accumulation of secondary metabolites and causes little oxidative injury, while high salinity impedes the secondary metabolites production and reduce antioxidative activities in LJF.

Both hierarchical clustering analysis and PLS-DA distinguish LJF samples from different salt stresses, of which compounds marked on the PLS-DA loading plot make it distinguish in different samples. Through GRA analysis, the best quality of LJF is obtained at 100 mM NaCl, followed by 0, 200, and the 300 mM. The results illustrate that the quality of LJF treated with 100 mM NaCl is optimal. The superiority of the control group to other salt stress groups also indicates that excessive salt is not conducive to the quality formation of LJF. Briefly, multivariate statistical analysis provides a basis for the complete differentiation of samples under different salt stresses and LJF quality evaluation.

Generally, salt stress tempestuously affects plant growth, physiological parameter, and metabolic process. However, plants can maintain osmotic balance, avoid oxidative damage, and keep normal growth through a series of adaptive responses. This experiment cultivated LJF under different salt stresses (0, 100, 200, and 300 mM NaCl) in the field to investigate the effects on the quality of LJF from the variations of morphology, photosynthesis, osmotic balance, antioxidant activity, bioactive constituents’ content. We find that the growth parameters, physiological indicators, and bioactive constituents content get the optimal values at 100 mM NaCl, indicating that salt stress could induce LJF remodeling transcriptional networks and metabolic pathways to regulate its growth and the accumulation of secondary metabolites. This will be verified with the multi-omics approach in the subsequent research. It will deepen acknowledge the mechanism of quality formation in LJF and provide a foundation for cultivation to improve desired characteristics.

## Conclusions

In the study, a reliable method combining morphology, physiology, and multiple bioactive constituents was proposed to evaluate the quality of LJF under different salt stresses (0, 100, 200, and 300 mM NaCl) based on UFLC-QTRAP-MS/MS and multivariate statistical analysis. The results demonstrated that LJF treated with 100 mM NaCl had the best quality; the increased accumulation of phenolic acids reduced oxidative damage; and low salt stress (100 mM) promote quality improvement, conversely, high salt (300 mM) inhibited that. Therefore, LJF cultivated in soil with 100 mM NaCl might be the optimal choice in the long run. Overall, this study sheds light on the effects of different salt stresses on the quality of LJF from the changes in morphology, physiology, and multiple bioactive constituents through field trials. The results might provide an integrated reference for the quality evaluation of other Chinese herbal medicines under salt stress and novel clues for improving the quality of medicinal plants in the pharmaceutical industry.

## Supplementary Information

Supplementary Information

## References

[1] Yang YQ, Guo Y (2018). Elucidating the molecular mechanisms mediating plant salt-stress responses. New Phytol..

[2] Dáder B (2019). Split green fluorescent protein as a tool to study infection with a plant pathogen, Cauliflower mosaic virus. PLoS ONE.

[3] Nasrollahi VN, Mirzaie-asl AM, Piri K, Nazeri S, Mehrabi R (2014). The effect of drought stress on the expression of key genes involved in the biosynthesis of triterpenoid saponins in liquorice (*Glycyrrhiza glabra*). Phytochemistry.

[4] Saito MA (2014). Multiple nutrient stresses at intersecting pacifi cocean biomes detected by protein biomarkers. Science.

[5] Spindel JE, McCouch SR (2016). When more is better: how data sharing would accelerate genomic selection of crop plants. New Phytol..

[6] Zhang JT, Zhang Y, Du YY, Chen SY, Tang HR (2011). Dynamic metabonomic responses of tobacco (*Nicotiana tabacum*) plants to salt stress. J. Proteome Res..

[7] Shang XF, Hu PN, Li MX (2011). *Lonicera japonica* Thunb.: thnopharmacology, phytochemistry and pharmacology of an important traditional Chinese medicine. J. Ethnopharmacol..

[8] He L (2013). Transcriptome analysis of buds and leaves using 454 pyrosequencing to discover genes associated with the biosynthesis of active ingredients in *Lonicera japonica* Thunb. PLoS ONE.

[9] Zhou Z (2015). Honeysuckle-encoded atypical microRNA2911 directly targets influenza A viruses. Cell Res..

[10] Wang LN, Jiang Q, Hu JH, Zhang YQ, Li J (2016). Research progress on chemical constituents of *Lonicerae japonicae flos*. Biomed. Res. Int..

[11] Slimestad R, Fossen T, Brede C (2020). Flavonoids and other phenolics in herbs commonly used in Norwegian commercial kitchens. Food Chem..

[12] Obied HK, Karuso P, Prenzler PD, Robards K (2007). Novel secoiridoids with antioxidant activity from Australian olive mill waste. J. Agric. Food Chem..

[13] Tian J, Che HL, Ha D, Wei YP, Zheng SY (2012). Characterization and anti-allergic effect of a polysaccharide from the flower buds of *Lonicera japonica*. Carbohydr. Polym..

[14] Sun CH (2010). Metabolomics study of the protective effects of *Lonicera japonica* extraction acute liver injury in dimethyl nitrosamine treated rats. J. Pharm. Biomed. Anal..

[15] Rawji KS (2016). Immunosenescence of microglia and macrophages: impact on the ageing central nervous system. Brain.

[16] Yang R (2019). Separation of five iridoid glycosides from Lonicerae Japonicae Flos using high-speed counter-current chromatography and their anti-inflammatory and antibacterial activities. Molecules.

[17] Yan K, Cui MX, Zhao SJ, Chen XB, Tang XL (2016). Salinity stress is beneficial to the accumulation of chlorogenic acids in honeysuckle (*Lonicera japonic*a Thunb.). Front. Plant Sci..

[18] Yan K, Zhao SJ, Bian LX, Chen XB (2017). Saline stress enhanced accumulation of leaf phenolics in honeysuckle (*Lonicera japonica* Thunb.) without induction of oxidative stress. Plant Physiol. Biochem..

[19] Cai ZC (2019). Quality evaluation of Lonicerae Japonicae Flos and Lonicerae Flos based on simultaneous determination of multiple bioactive constituents combined with multivariate statistical analysis. Phytochem. Anal..

[20] contribution to the overall antioxidant activity (2012). Seo, O.N. *et al*. Determination of polyphenol components of *Lonicera japonica* Thunb. using liquid chromatography–tandem mass spectrometry. Food Chem..

[21] Gosa SC, Lupo Y, Moshelion M (2019). Quantitative and comparative analysis of whole-plant performance for functional physiological traits phenotyping: new tools to support pre-breeding and plant stress physiology studies. Plant Sci..

[22] Shao YH (2015). Effect of salt treatment on growth, isoenzymes and metabolites of Andrographis paniculata (Burm. F.) Nees. Acta Physiol. Plant..

[23] Yan K, Wu CW, Zhang LH, Chen XB (2015). Contrasting photosynthesis and photoinhibition in tetraploid and its autodiploid honeysuckle (*Lonicera japonica* Thunb.) under salt stress. Front. Plant Sci..

[24] Lichtenthaler HK, Wellburn AR (1985). Determination of total carotenoids and chlorophylls a and b of leaf in different solvents. Biochem. Soc. Trans..

[25] Wang XH, Huang JL (2015). Principles and techniques of plant physiological biochemical experiment.

[26] Zeng JW, Chen AM, Li DD (2013). Effects of salt stress on the growth, physiological responses, and glycoside contents of stevia rebaudiana Bertoni. J. Agric. Food Chem..

[27] Chen CH, Wang CC, Liu ZX (2018). Variations in physiology and multiple bioactive constituents under salt stress provide insight into the quality evaluation of apocyni veneti folium. Int. J. Mol. Sci..

[28] Wu FB, Zhang GP, Dominy P (2003). Four barley genotypes respond differently to cadmium: lipid peroxidation and activities of antioxidant capacity. Environ. Exp. Bot..

[29] Cai ZC (2019). Comparison of multiple bioactive constituents in the flower and the caulis of *Lonicera japonica* based on UFLC-QTRAP-MS/MS combined with multivariate statistical analysis. Molecules.

[30] Chen CH (2019). A strategy for quality evaluation of salt-treated Apocyni Veneti Folium and discovery of efficacy-associated markers by fingerprint-activity relationship modeling. Sci. Rep..

[31] Imada S, Yamanaka N, Tamai S (2009). Effects of salinity on the growth, Na partitioning, and Na dynamics of a salt-tolerant tree, Populus alba L. J. Arid Environ..

[32] Cheng SJ, Tang DQ, Miller WB, Shi YM (2018). Evaluation of salinity tolerance in honeysuckle (*Lonicera japonica*) using growth, ion accumulation, lipid peroxidation, and non-enzymatic and enzymatic antioxidants system criteria. J. Hortic. Sci. Biotechnol..

[33] Meloni DA, Gulotta MR, Martinez CA, Oliva MA (2004). The effects of salt stress on growth, nitrate reduction and proline and glycine betaine accumulation in *Prosopis alba*. Braz. J. Plant Physiol..

[34] Kumar S, Beena AS, Awana M, Singh A (2017). Physiological, biochemical, epigenetic and molecular analyses of wheat (Triticum aestivum) genotypes with contrasting salt tolerance. Front. Plant Sci..

[35] Khan MA, Ungar IA, Showalter AM (2000). The effect of salinity on the growth, water status, and ion content of a leaf succulent perennial halophyte, *Suaeda fruticosa* (L.) Forssk. J. Arid Environ..

[36] Bavei V, Shiran B, Arzani A (2011). Evaluation of salinity tolerance in sorghum (*Sorghum bicolor* L.) using ion accumulation, proline and peroxidase criteria. J. Plant Growth Regul..

[37] Sergio L (2012). Effect of salt stress on growth parameters, enzymatic antioxidant system, and lipid peroxidation in wild chicory (*Cichorium intybus* L.). Acta Physiol. Plant..

[38] Mansour MMF, Ali EF (2017). Evaluation of proline functions in saline conditions. Phytochemistry.

[39] Liang MH, Liang ZC, Chen HH, Jiang GJ (2019). The bifunctional identification of both lycopene beta- and epsilon-cyclases from the lutein-rich Dunaliella bardawil. Enzyme Microb. Technol..

[40] Bendaly A (2016). Physiological and leaf metabolome changes in the xerophyte species Atriplex halimus induced by salinity. Plant Physiol. Biochem..

[41] Munns R, Tester M (2008). Mechanisms of salinity tolerance. Annu. Rev. Plant Biol..

[42] Nabi RBS (2019). Nitric oxide regulates plant responses to drought, salinity, and heavy metal stress. Environ. Exp. Bot..

[43] Mishra A, Patel MK, Jha B (2015). Non-targeted metabolomics and scavenging activity of reactive oxygen species reveal the potential of Salicornia brachiata as a functional food. J. Funct. Foods.

[44] Niu X, Bressan RA, Hasegawa PM, Pardo JM (1995). Ion homeostasis in NaCl stress environments. Plant Physiol..

[45] Li YQ, Kong DX, Fu Y, Sussman RM, Wu H (2020). The effect of developmental and environmental factors on secondary metabolites in medicinal plants. Plant J. Physiol. Biochem..

[46] Niggeweg R, Michael AJ, Martin C (2004). Engineering plants with increased levels of the antioxidant chlorogenic acid. Nat. Biotechnol..

[47] Kong D (2017). Correlation between the dynamic accumulation of the main effective components and their associated regulatory enzyme activities at different growth stages in *Lonicera japonica*Thunb. Ind. Crops Prod..

[48] Abrol E, Vyas D, Koul S (2012). Metabolic shift from secondary metabolite production to induction of anti-oxidative enzymes during NaCl stress in Swertia chirata Buch.-Ham. Acta Physiol. Plant..

