# Supplementary material for: Variations in morphology, physiology, and multiple bioactive constituents of Lonicerae Japonicae Flos under salt stress
Source: Sci Rep. 2021 Feb 16;11:3939. doi: 10.1038/s41598-021-83566-6 (PMC7887249; doi:10.1038/s41598-021-83566-6)
Supplement: Supplementary file 1 — Supplementary Information [file 41598_2021_83566_MOESM1_ESM.pdf]

Variations in morphology, physiology, and multiple bioactive constituents of Lonicerae Japonicae Flos under salt stress

Authors:

Zhichen Cai [1], caizhichen2008@126.com

Xunhong Liu [1, 2, 3] *, liuxunh1959@163.com

Huan Chen [1], 2495475801@qq.com

Rong Yang [1], yr2507878828@163.com

Jiajia Chen [1], 513923212@qq.com

Lisi Zou [1], zlstcm@126.com

Chengcheng Wang [1], ccw199192@163.com

Jiali Chen [1], 18994986833@163.com

Mengxia Tan [1], 18816250751@163.com

Yuqi Mei [1], 18260028173@163.com

Lifang Wei [1], weilifang1995@yeah.net

1 *College of Pharmacy, Nanjing University of Chinese Medicine, Nanjing 210023, China*

2 *Collaborative Innovation Center of Chinese Medicinal Resources Industrialization, Nanjing 210023, China*

3 *National and Local Collaborative Engineering Center of Chinese Medicinal Resources Industrialization and Formulae Innovative Medicine, Nanjing 210023, China*

* Corresponding authors.

Supplemental Figures

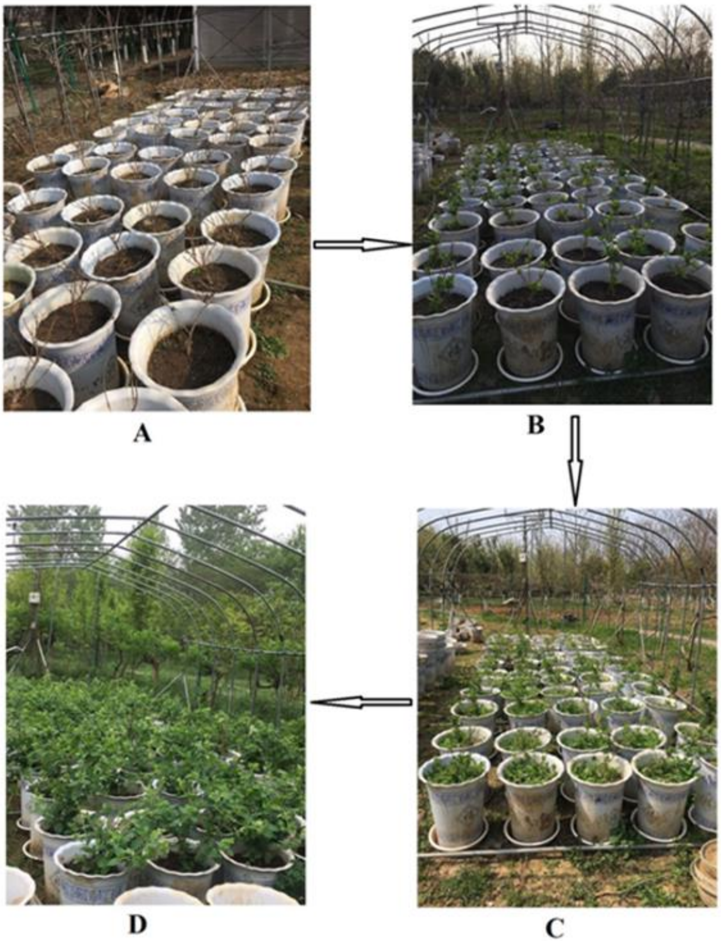

Fig. S1. Leaf (A) and flower bud (B) under 0, 100, 200, 300 mM NaCl treatment during the plants were the blooming stage

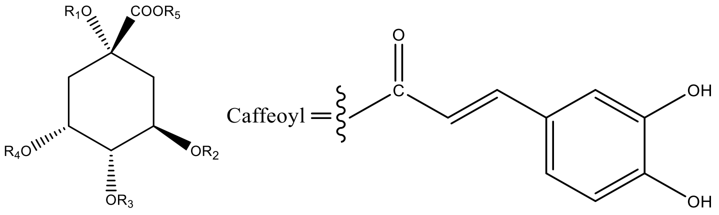

Quinic acid: R_1=H, R_2=H, R_3=H, R_4=H, R_5=H

Chlorogenic acid: R_1=H, R_2=Caffeoyl, R_3=H, R4=H, R_5=H

Neochlorogenic acid: R_1=H, R_2=H, R3=H, R_4=Caffeoyl, R_5=H

Cryptochlorogenic acid: R_1=H, R_2=H, R_3=Caffeoyl, R_4=H, R_5=H

1,3-O-dicaffeoylquinic acid: R_1=H, R_2=Caffeoyl, R_3=H, R_4=H, R_5=Caffeoyl

3,5-O-dicaffeoylquinic acid: R_1=H, R_2=Caffeoyl, R_3=H, R_4=Caffeoyl, R_5=H

3,4-O-dicaffeoylquinic acid: R_1=H, R_2=Caffeoyl, R_3=Caffeoyl, R_4=H, R_5=H

4,5-O-dicaffeoylquinic acid: R_1=H, R_2=H, R_3=Caffeoyl, R_4=Caffeoyl, R_5=H

4,5-O-dicaffeoylquinic acid methyl ester: R_1=H, R_2=H, R_3=Caffeoyl, R_4=Caffeoyl, R_5=CH_3

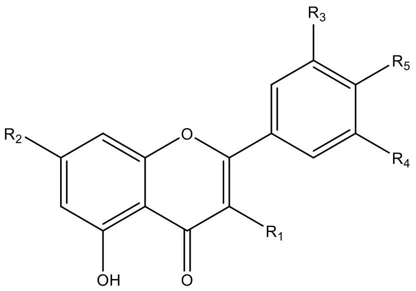

Diosmetin: R_1=H, R_2=OH, R_3=OCH_3, R_4=H, R_5=OH

Rutin: R_1=O-rutinose, R_2=OH, R_3=OH, R_4=H, R_5=OH

Isoquercetin: R_1=O-Glc, R_2=OH, R_3=OH, R_4=H, R_5=OH

Hyperoside: R_1=O-Gal, R_2=OH, R_3=OH, R_4=H, R_5=OH

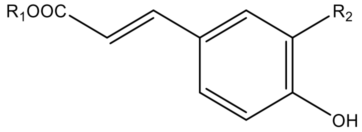

Caffeic acid: R_1=H, R_2=OH

Protocatechuic acid: R_1=H, R_2=OH

Ferulic acid: R_1=H, R_2=OCH_3

Luteoloside: R_1=H, R_2=Glc, R_3=H, R_4=OH, R_5=OH

Luteolin: R_1=H, R_2=OH, R_3=OH, R_4=H, R_5=OH

Lonicerin: R_1=H, R_2=L-mannose and D-Glc, R_3=OH, R_4=H, R_5=OH

Rhoifolin: R_1=H, R_2= L-mannose and D-Glc, R_3=H, R_4=H, R_5=OH

Apigenin: R_1=H, R_2=OH, R_3=H, R_4=H, R_5=OH

Kaempferol-3-rutinoside: R_1=O-Gal, R_2=OH, R_3=H, R_4=H, R_5=OH

Kaempferol: R_1=OH, R_2=OH, R_3=H, R_4=H, R_5=OH

Astragalin: R_1=O-Glc, R_2=OH, R_3=H, R_4=H, R_5=OH

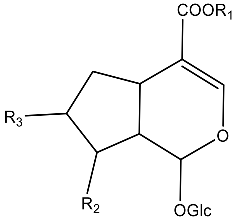

Loganin: R_1=CH_3, R_2=CH_3, R_3=OH

Loganin acid: R_1=H, R_2=CH_3, R_3=OH

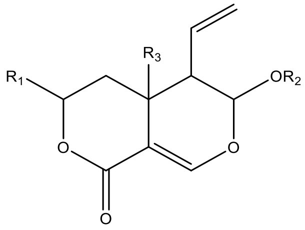

Sweroside: R_1=H, R_2=Glc, R_3=H

Secologanic acid: R_1=OH, R_2=Glc, R_3=H

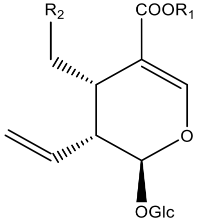

Secoxyloganin: R_1=CH_3, R_2=COOH

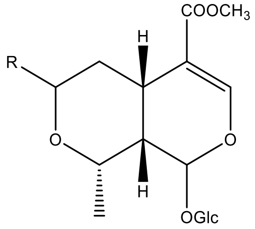

Morroniside: R=OH

Fig. S2. The structure of 47 bioactive components

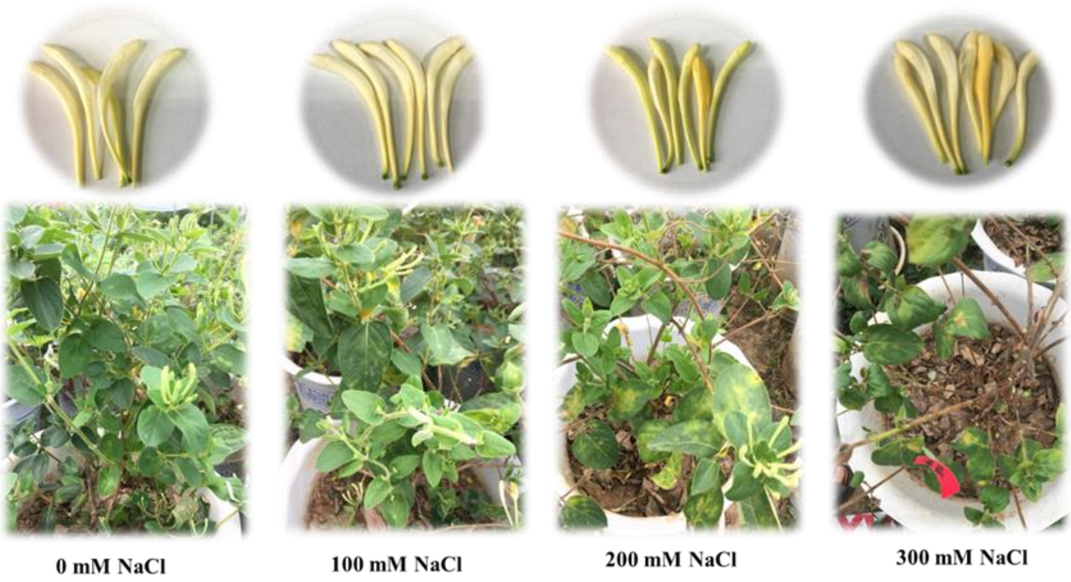

Fig. S3. Phenotype profiles of representative *L*. japonica

Supplemental Tables

Table S1. Chemicals and reagents information

Chemicals or Reagents	CAS no.	Source
Apigenin	520-36-5	Chengdu Chroma Biotechnology Co. Ltd (Sichuan, China)
Diosmetin	520-34-3	Chengdu Chroma Biotechnology Co. Ltd (Sichuan, China)
Kaempferol	520-18-3	Chengdu Chroma Biotechnology Co. Ltd (Sichuan, China)
Kaempferol-3-O-rutinoside	17650-84-9	Chengdu Chroma Biotechnology Co. Ltd (Sichuan, China)
Sweroside	14215-86-2	Chengdu Chroma Biotechnology Co. Ltd (Sichuan, China)
Quinic acid	77-95-2	Control of Pharmaceutical and Biological Products (Beijing, China)
Rutin	153-18-4	Control of Pharmaceutical and Biological Products (Beijing, China)
Astragalin	480-10-4	Control of Pharmaceutical and Biological Products (Beijing, China)
Hyperoside	482-36-0	Control of Pharmaceutical and Biological Products (Beijing, China)
Isoquercitrin	482-35-9	Control of Pharmaceutical and Biological Products (Beijing, China)
1,3-O-dicaffeoylquinic acid	19870-46-3	Chengdu Prefa Technology Development Co. Ltd (Sichuan, China)
Isochlorogenic acid C	57378-72-0	Chengdu Prefa Technology Development Co. Ltd (Sichuan, China)
Protocatechuic acid	99-50-3	Chengdu Prefa Technology Development Co. Ltd (Sichuan, China)
4,5-O-dicaffeoylquinic acid methyl ester	114637-83-1	Liangwei Chemical Reagent Co. Ltd (Nanjing, China)
Luteoloside	5373-11-5	Liangwei Chemical Reagent Co. Ltd (Nanjing, China)
Luteolin	491-70-3	Liangwei Chemical Reagent Co. Ltd (Nanjing, China)
Rhoifolin	17306-46-6	Liangwei Chemical Reagent Co. Ltd (Nanjing, China)
Lonicerin	25694-72-8	Liangwei Chemical Reagent Co. Ltd (Nanjing, China)
Secologanic acid	60077-46-5	Liangwei Chemical Reagent Co. Ltd (Nanjing, China)

Loganin acid	22255-40-9	Liangwei Chemical Reagent Co. Ltd (Nanjing, China)
Morroniside	25406-64-8	Liangwei Chemical Reagent Co. Ltd (Nanjing, China)
Secoxyloganin	58822-47-2	Nanjing Jingzhu Biotechnology Co. Ltd (Nanjing, China)
Chlorogenic acid	327-97-9	Shanghai Yuanye Biotechnology Co. Ltd (Shanghai, China)
Neochlorogenic acid	906-33-2	Shanghai Yuanye Biotechnology Co. Ltd (Shanghai, China)
Cryptochlorogenic acid	905-99-7	Shanghai Yuanye Biotechnology Co. Ltd (Shanghai, China)
Isochlorogenic acid A	2450-53-5	Shanghai Yuanye Biotechnology Co. Ltd (Shanghai, China)
Isochlorogenic acid B	14534-61-3	Shanghai Yuanye Biotechnology Co. Ltd (Shanghai, China)
Ferulic acid	1135-24-6	Shanghai Yuanye Biotechnology Co. Ltd (Shanghai, China)
Caffeic acid	331-39-5	Shanghai Yuanye Biotechnology Co. Ltd (Shanghai, China)
Loganin	18524-94-2	Shanghai Yuanye Biotechnology Co. Ltd (Shanghai, China)
L-alanine	56-41-7	Shanghai Yuanye Biotechnology Co. Ltd (Shanghai, China)
L-serine	56-45-1	Shanghai Yuanye Biotechnology Co. Ltd (Shanghai, China)
L-proline	147-85-3	Shanghai Yuanye Biotechnology Co. Ltd (Shanghai, China)
L-valine	72-18-4	Shanghai Yuanye Biotechnology Co. Ltd (Shanghai, China)
L-threonine	72-19-5	Shanghai Yuanye Biotechnology Co. Ltd (Shanghai, China)
L-isoleucine	73-32-5	Shanghai Yuanye Biotechnology Co. Ltd (Shanghai, China)
L-leucine	61-90-5	Shanghai Yuanye Biotechnology Co. Ltd (Shanghai, China)
L-aspartic acid	56-84-8	Shanghai Yuanye Biotechnology Co. Ltd (Shanghai, China)
L-glutanmate	138-18-1	Shanghai Yuanye Biotechnology Co. Ltd (Shanghai, China)
L-lysine	56-87-1	Shanghai Yuanye Biotechnology Co. Ltd (Shanghai, China)
L-histidine	71-00-1	Shanghai Yuanye Biotechnology Co. Ltd (Shanghai, China)
L-phenylalanine	63-91-2	Shanghai Yuanye Biotechnology Co. Ltd (Shanghai, China)
L-arginine	74-79-3	Shanghai Yuanye Biotechnology Co. Ltd (Shanghai, China)
Cytidine	65-46-3	Shanghai Yuanye Biotechnology Co. Ltd (Shanghai, China)
Uridine	58-96-8	Shanghai Yuanye Biotechnology Co. Ltd (Shanghai, China)
Adenosine	58-61-7	Shanghai Yuanye Biotechnology Co. Ltd (Shanghai, China)
Inosine	58-63-9	Shanghai Yuanye Biotechnology Co. Ltd (Shanghai, China)

Table S2. Retention time and related MS data of the 47 compounds.

No.	Compounds	Formula	t_R (min)	MV	MRM (percursor→product)	DP	CE
1	L-Alanine	$C_3H_7NO_2$	1.23	89.09	90.06/44.02	100	10
2	L-Serine	$C_3H_7NO_3$	1.22	105.09	106.05/59.99	100	8
3	L-Proline	$C_5H_9NO_2$	1.34	115.13	116.07/70.02	68	10
4	L-valine	$C_5H_{11}NO_2$	1.83	117.15	118.09/72.06	100	10
5	L-Threonine	$C_4H_9NO_3$	1.25	119.12	120.07/74	100	20
6	L-Isoleucine	$C_6H_{13}NO_2$	3.19	131.17	132.1/86.05	64	10
7	L-Leucine	$C_6H_{13}NO_2$	3.56	131.17	132.1/86.05	100	16
8	L-aspartic acid	$C_4H_7NO_4$	1.23	133.1	134.05/87.96	59	10
9	L-Glutamate	$C_5H_7NO_4$	1.22	145.11	147.08/83.92	100	16
10	L-lysine	$C_6H_{14}N_2O_2$	1.22	146.19	147.11/83.91	100	14
11	L-Histidine	$C_6H_9N_3O_2$	1.12	155.15	156.08/110.03	100	16

12	L-Phenylalanine	$C_9H_{11}NO_2$	6.78	165.19	166.1/120.05	100	14
13	L-Arginine	$C_6H_{14}N_4O_2$	1.22	174.2	175.12/70.02	100	18
14	Cytidine	$C_9H_{13}N_3O_5$	1.49	243.22	244.09/112	61	10
15	Uridine	$C_9H_{12}N_2O_6$	2.37	244.2	244.896/113	10	13
16	Adenosine	$C_{10}H_{13}N_5O_4$	2.81	267.24	268.1/136.07	86	23
17	Inosine	$C_{10}H_{12}N_4O_5$	3.5	268.22	269/137.07	46	15
18	Chlorogenic acid	$C_{16}H_{18}O_9$	11.6	354.31	305.01/125	-35	-20
19	Neochlorogenic acid	$C_{16}H_{18}O_9$	11.4	354.31	305.01/125	-80	-26
20	Cryptochlorogenic acid	$C_{16}H_{18}O_9$	13.23	354.31	305.01/125	-95	-20
21	Isochlorogenic A	$C_{25}H_{24}O_{12}$	18.62	516.45	515.1/191	-85	-22
22	Isochlorogenic B	$C_{25}H_{24}O_{12}$	18.01	516.45	514.989/353	-80	-26
23	Isochlorogenic C	$C_{25}H_{24}O_{12}$	20.1	516.45	515.1/191	-75	-24
24	1,3-O-dicaffeoylquinic acid	$C_{25}H_{24}O_{12}$	14.1	516.45	514.980/190.979	-95	-24
25	Caffeic acid	$C_9H_8O_4$	12.2	180.16	179.03/134.6	-125	-20
26	Quinic acid	$C_7H_{12}O_6$	11.5	192.17	191.099/84.981	-195	-28
27	Protocatechuic acid	$C_7H_6O_4$	7.76	154.12	152.9/109	-85	-16
28	Ferulic acid	$C_{10}H_{10}O_4$	19.32	194.18	193.017/134	-50	-10
29	4,5-O-dicaffeoylquinic acid methyl ester	$C_{26}H_{26}O_{12}$	20.3	530.47	529.194/135.001	-85	-42
30	Rutin	$C_{27}H_{30}O_{16}$	16.3	610.52	609.06/300	-245	-46
31	Hyperoside	$C_{21}H_{20}O_{12}$	16.8	464.38	463.003/299.9	-160	-36
32	Luteoloside	$C_{21}H_{20}O_{11}$	16.9	448.38	447.117/284.963	-300	-36
33	Luteolin	$C_{15}H_{10}O_6$	21.5	286.24	285.086/132.980	-170	-40
34	Rhoifolin	$C_{27}H_{30}O_{14}$	18.02	578.52	577.185/268.958	-65	-46
35	Diosmetin	$C_{16}H_{12}O_6$	24.93	300.26	298.938/283.929	-215	-30
36	Apigenin	$C_{15}H_{10}O_5$	24.24	270.24	268.8/116.9	-129	-40
37	Kaempferol	$C_{15}H_{10}O_6$	21.44	286.24	285.0/116.9	-120	-36
38	Astragalin	$C_{21}H_{20}O_{11}$	16.91	448.38	447.1/283.9	-100	-36
39	Lonicerin	$C_{27}H_{30}O_{15}$	16.9	594.52	593.146/283.984	-200	-54
40	Kaempferol-3-O-rutinoside	$C_{27}H_{30}O_{15}$	16.99	594.52	595/287.2	36	25
41	Isoquercitrin	$C_{21}H_{20}O_{12}$	16.8	464.38	463.015/300	-180	-36
42	Sweroside	$C_{16}H_{22}O_9$	13.7	358.34	357.213/124.985	-65	-20
43	Secologanic acid	$C_{16}H_{22}O_{10}$	12.2	376.36	357.107/212.956	-170	-22
44	Loganin acid	$C_{16}H_{24}O_{10}$	10.8	376.36	375.107/212.956	-170	-22
45	Secoxyloganin	$C_{17}H_{24}O_{11}$	15.3	404.37	403.219/120.973	-135	-32
46	Loganin	$C_{17}H_{26}O_{10}$	13.2	390.38	389.262/226.980	-40	-12
47	Morroniside	$C_{17}H_{26}O_{11}$	11.3	406.38	405.235/243	-100	-14

Table S3. Contents of 47 bioactive components in LJF under salt stress (Control group: S1-5, low salt stress: S6-10, medium salt stress: S11-15, high salt stress: S16-20).

No.	Compound	S1	S2	S3	S4	S5	S6	S7	S8	S9	S10	S11	S12	S13	S14	S15	S16	S17	S18	S19	S20
1	L-Alanine	7614.91	6478.55	4378.55	6342.18	5769.45	10051.27	10960.36	9596.73	10324.00	9869.45	10960.36	8887.64	8369.45	8442.18	7942.18	10414.91	7514.91	8205.82	8805.82	7769.45
2	L-Serine	1901.86	1393.71	1022.38	2406.74	2009.35	4410.00	4442.57	4410.00	4442.57	4344.85	3335.08	3563.09	3367.65	3465.37	3530.52	3367.65	3181.99	3142.90	3335.08	3191.76
3	L-Proline	4518.97	4612.99	4442.05	4313.85	4382.22	4014.70	3741.20	4177.09	4160.00	3954.87	4254.02	4219.83	3971.97	4330.94	4493.33	4057.44	4074.53	4108.72	4536.07	3963.42
4	L-Valine	14762.02	8741.82	9085.25	16075.15	17933.74	19408.48	16721.62	20782.22	18458.99	18923.64	17509.49	20378.18	19307.47	17751.92	19125.66	18075.15	17469.09	17368.08	18701.41	16236.77
5	L-Threonine	3051.48	1804.09	7426.48	2921.28	3077.53	4666.07	3962.94	4483.78	4666.07	4692.11	4796.28	4145.23	3884.82	4041.07	3650.44	3832.73	0.00	3207.73	3363.98	2921.28
6	L-Isoleucine	1786.87	1620.20	660.20	2213.53	2400.20	0.00	5400.20	0.00	4880.20	5480.20	0.00	5860.20	6000.20	5600.20	5540.20	4946.87	5026.87	0.00	4853.53	5206.87
7	L-Leucine	1593.61	1515.54	593.61	1928.18	2073.16	0.00	4114.05	0.00	3779.48	3705.13	0.00	4337.10	3891.00	3853.83	3779.48	3891.00	3482.08	3965.35	3612.19	3589.89
8	L-Aspartic acid	1636.11	1091.67	1025.00	1463.89	1841.67	2402.78	2563.89	2447.22	2419.44	2441.67	2469.44	2213.89	2025.00	2086.11	2075.00	2025.00	1580.56	1680.56	1747.22	1625.00
9	L-Glutamate	32945.85	24281.59	16592.06	58577.62	47386.28	83126.35	87819.49	86014.44	88541.52	86555.96	83306.86	78974.73	75906.14	74823.10	69949.46	63631.77	50274.37	54967.51	56772.56	50815.88
10	L-Lysine	30301.81	23833.51	15295.34	51129.75	40909.83	66524.32	69887.84	67688.62	68982.28	67947.35	67171.15	64971.93	63678.27	63031.44	59667.92	55140.10	44273.35	48413.07	47895.60	46472.57
11	L-Histidine	540.04	476.21	432.14	540.04	435.18	746.73	730.01	781.68	743.69	761.93	649.47	581.08	590.19	676.82	647.95	550.68	505.09	546.12	517.25	570.44
12	L-Phenylalanine	1663.87	1591.70	736.03	1076.24	2272.11	3442.22	3741.19	3803.04	3344.28	3452.53	2983.45	3411.29	3163.87	3230.88	3179.33	2550.46	2498.92	2421.60	2457.68	2488.61
13	L-Arginine	654.03	629.19	324.50	595.64	982.89	1942.62	1848.66	1929.19	1868.79	1935.91	2405.70	2264.77	2412.42	2439.26	2727.85	1633.89	1687.58	1687.58	1815.10	1607.05
14	Cytidine	108.16	135.77	97.70	78.03	189.33	161.44	139.12	168.41	160.04	139.12	240.93	279.98	245.12	320.43	300.91	264.64	235.35	284.17	238.14	224.20
15	Uridine	3949.90	3845.73	7359.62	6484.62	4317.95	1463.78	2727.67	1755.45	1804.06	2269.34	1824.90	3401.28	2672.12	3373.51	3817.95	3741.56	2769.34	2067.95	3755.45	3366.56
16	Adenosine	0.00	424.79	0.00	0.00	0.00	0.00	0.00	206.27	234.42	0.00	0.00	0.00	277.39	0.00	220.35	281.09	0.00	292.94	230.72	281.09
17	Inosine	2283.02	3301.01	2372.95	2247.05	2408.92	1851.37	2247.05	2024.03	2088.78	1844.17	2157.12	2376.55	2254.24	2362.16	2038.42	2646.33	2135.54	2369.35	2347.77	2585.18
18	Chlorogenic acid	20468.05	19126.82	21059.76	20073.57	19639.64	19107.10	16187.97	16306.31	19166.27	21059.76	22637.67	19028.21	19876.33	20665.29	20270.81	18752.07	18061.74	17726.43	17864.50	19304.34
19	Neochlorogenic acid	2122.12	2067.93	1728.03	1836.40	2058.08	3831.48	3989.11	2979.26	2831.48	2742.81	1910.30	3393.05	3402.91	2728.03	3309.31	3668.92	3072.86	3397.98	3358.57	3240.34
20	Cryptochlorogenic acid	8812.50	8587.09	7173.16	7623.98	8546.11	15923.16	16578.89	12378.07	11763.32	11394.47	7931.35	14099.39	14140.37	11332.99	13751.02	15246.93	12767.42	14119.88	13955.94	13464.14
21	Isochlorogenic A	7360.76	7171.43	5983.82	6362.48	7137.01	13333.22	13883.99	10355.59	9839.24	9529.43	6620.65	11801.38	11835.80	9477.80	11508.78	12765.23	10682.62	11818.59	11680.90	11267.81
22	Isochlorogenic B	84.42	112.94	134.90	110.39	96.55	47.65	52.86	53.96	52.26	54.08	49.83	47.28	39.64	45.10	43.16	48.98	49.10	50.80	53.59	58.69
23	Isochlorogenic C	183880.66	398283.95	413921.81	418037.04	408983.54	397872.43	336967.08	339436.21	399107.00	409806.58	198283.95	193345.68	187172.84	191699.59	187584.36	189641.98	178530.86	167419.75	181411.52	185938.27
24	1,3-O-dicaffeoylquinic acid	4731.12	5307.59	5337.00	5160.53	3613.47	672.29	737.00	760.53	637.00	672.29	412.29	479.35	442.88	451.12	431.12	496.41	427.59	451.71	464.06	461.71

25	Caffeic acid	0.00	0.00	0.00	12.14	17.17	20.09	18.82	20.31	19.24	16.47	14.62	16.95	18.78	13.79	18.45	21.61	21.61	22.48	21.61	23.57
26	Quinic acid	160.69	170.59	152.77	129.01	104.36	196.34	226.04	228.02	240.89	242.87	208.22	215.15	202.28	238.91	184.46	184.46	185.45	183.47	230.99	208.22
27	Protocatechuic acid	153.88	148.56	160.79	136.32	213.98	309.73	354.94	347.49	309.73	324.62	645.90	768.24	688.45	688.45	672.49	111.86	109.73	109.20	115.05	116.64
28	Ferulic acid	304604.98	290014.23	284676.16	287523.13	270441.28	279338.08	282185.05	281829.18	282896.80	273288.26	292149.47	281117.44	270085.41	252647.69	255494.66	308875.44	282540.93	285743.77	290014.23	286811.39
29	4,5-O-dicaffeoylquinic acid methyl ester	3824.59	3423.06	3518.66	3078.89	2581.76	4321.72	4914.46	5029.18	4379.08	4493.80	5602.79	6042.56	5526.31	5526.31	5354.23	4034.91	3824.59	3920.19	4054.03	4034.91
30	Rutin	19487.08	17028.75	17320.42	18570.42	21153.75	14653.75	15320.42	17403.75	15695.42	16237.08	12820.42	13403.75	13070.42	12403.75	13737.08	15570.42	16112.08	15862.08	16153.75	16403.75
31	Hyperoside	10046.45	9572.51	10520.38	10946.92	11705.21	6918.48	7771.56	7771.56	6776.30	7013.27	4297.63	4425.59	4600.95	3785.78	4581.99	6302.37	6491.94	6397.16	6065.40	6634.12
32	Luteoloside	1505.09	1696.32	1961.23	1448.95	2189.30	2382.28	2926.14	2733.16	2382.28	2487.54	2908.60	3470.00	2680.53	2978.77	2926.14	3084.04	3189.30	3206.84	3294.56	3241.93
33	Luteolin	4513.08	4077.11	4322.34	4267.85	5017.17	4322.34	4676.57	4744.69	4444.96	4553.95	3559.40	4090.74	3722.89	4077.11	3668.39	4145.23	4077.11	3954.50	4444.96	4172.48
34	Rhoifolin	16843.75	14827.62	14928.43	17650.20	20775.20	18456.65	20170.36	19565.52	17448.59	17952.62	12912.30	14021.17	14222.78	12408.27	14222.78	20775.20	20573.59	21380.04	19363.91	20674.40
35	Diosmetin	0.00	0.00	0.00	0.00	0.00	1080.61	1232.98	0.00	924.75	0.00	0.00	0.00	0.00	0.00	0.00	0.00	814.41	1126.15	847.69	886.22
36	Apigenin	4.40	1.37	4.31	2.11	16.11	7.48	8.58	14.02	10.44	10.82	5.52	7.57	2.16	10.00	7.00	23.70	26.17	27.69	25.22	28.63
37	Kaempferol	41.85	53.28	72.71	46.28	52.42	41.64	47.71	48.35	42.35	44.64	49.92	58.49	46.92	52.56	53.64	51.56	51.92	54.35	54.85	55.35
38	Astragalin	29.78	30.38	36.92	25.87	45.12	40.16	46.02	45.42	41.21	41.36	48.43	56.32	47.00	51.06	51.74	53.47	54.22	53.09	54.89	52.94
39	Lonicerin	385.34	458.61	460.05	513.21	449.99	283.33	304.88	274.71	228.73	243.10	283.33	205.74	188.50	192.81	179.88	406.89	284.76	379.59	277.58	314.94
40	Kaempferol-3-O-rutinoside	3207.74	2892.42	3081.61	3063.59	3568.10	3090.62	3351.88	3351.88	3162.69	3252.78	2487.02	2946.48	2721.25	2775.31	2667.20	2982.51	3009.54	2802.33	3171.70	3099.63
41	Isoquercitrin	1112.82	986.38	1038.10	1178.91	1328.33	1084.08	1164.54	1219.14	1150.17	1204.77	891.55	1104.20	1017.99	1029.48	1017.99	1043.85	1144.43	1084.08	1170.29	1164.54
42	Sweroside	367.95	414.25	290.79	408.08	435.85	2195.11	2611.78	2670.42	2275.36	1991.41	404.99	580.92	500.67	451.29	454.37	914.25	889.56	877.21	852.52	821.66
43	Secologanic acid	24791.78	17873.97	10682.19	12257.53	26435.62	41504.11	41641.10	46504.11	44380.82	47052.05	42531.51	38490.41	35956.16	36915.07	35956.16	37120.55	34860.27	34449.32	36915.07	36367.12
44	Loganin acid	229.69	297.66	161.72	477.34	245.31	1183.59	761.72	777.34	1066.41	640.63	314.84	988.28	357.81	855.47	417.97	1511.72	902.34	1050.78	1277.34	1324.22
45	Loganin	33618.83	41151.30	34203.25	34917.53	15307.14	14982.47	18424.03	19917.53	14722.73	15177.27	19982.47	16735.71	13813.64	13878.57	13294.16	24138.31	21995.45	23813.64	23553.90	19982.47
46	Secoxyloganin	125667.72	123777.95	126455.12	115274.02	115588.98	101730.71	101258.27	103148.03	100313.39	106297.64	76376.38	73541.73	70234.65	70549.61	70549.61	102045.67	93541.73	93069.29	97321.26	96848.82
47	Morroniside	17984.59	15979.57	13473.31	14099.87	15603.63	28761.53	31267.79	31894.36	28260.28	30014.66	23874.31	26255.26	24626.19	23749.00	24500.88	38160.03	37408.15	37032.21	38410.65	38911.90
